# The EZH2-H3K27me3 axis modulates aberrant transcription and apoptosis in cyclophosphamide-induced ovarian granulosa cell injury

**DOI:** 10.1038/s41420-023-01705-6

**Published:** 2023-11-14

**Authors:** Yingyan Chen, Leilei Ai, Yingyi Zhang, Xiang Li, Shiqian Xu, Weijie Yang, Jiamin Jin, Yerong Ma, Zhanhong Hu, Yinli Zhang, Yan Rong, Songying Zhang

**Affiliations:** 1 Department of Obstetrics and Gynecology, Sir Run Run Shaw Hospital, School of Medicine, Zhejiang University, Hangzhou, China; 2 Key Laboratory of Reproductive Dysfunction Management of Zhejiang Province; Zhejiang Provincial Clinical Research Center for Obstetrics and Gynecology, Hangzhou, China

**Keywords:** Gene silencing, Apoptosis

## Abstract

Chemotherapy-induced ovarian damage and infertility are significant concerns for women of childbearing age with cancer; however, the underlying mechanisms are still not fully understood. Our study has revealed a close association between epigenetic regulation and cyclophosphamide (CTX)-induced ovarian damage. Specifically, CTX and its active metabolite 4-hydroperoxy cyclophosphamide (4-HC) were found to increase the apoptosis of granulosa cells (GCs) by reducing EZH2 and H3K27me3 levels, both in vivo and in vitro. Furthermore, RNA-seq and CUT&Tag analyses revealed that the loss of H3K27me3 peaks on promoters led to the overactivation of genes associated with transcriptional regulation and apoptosis, indicating that stable H3K27me3 status could help to provide a safeguard against CTX-induced ovarian damage. Administration of the H3K27me3-demethylase inhibitor, GSK-J4, prior to CTX treatment could partially mitigate GC apoptosis by reversing the reduction of H3K27me3 and the aberrant upregulation of specific genes involved in transcriptional regulation and apoptosis. GSK-J4 could thus potentially be a protective agent for female fertility when undergoing chemotherapy. The results provide new insights into the mechanisms for chemotherapy injury and future clinical interventions for fertility preservation.

## Introduction

Over the last two decades there have been significant advances in the development of early diagnosis methods and regular treatments for cancers, and consequently, the long-term survival rates of patients with malignant tumors have greatly increased [1]. Current conventional oncotherapies include surgery, radiotherapy and chemotherapy. All of these therapies, however, will inevitably impair the reproductive system and fertility of the patients [2]. There is thus a need for fertility protection and preservation strategies, especially for children, adolescents, and young female patients. Clinical treatments to preserve female fertility include adjuvant medicine, assisted reproduction technologies for oocyte, embryo, ovarian cortex cryopreservation, and the emerging stem cell therapies [3, 4]. However, the effectiveness of the above strategies and subsequent pregnancy rates are not satisfactory. Exploring the mechanisms of ovarian damage in relation to oncotherapy may shed light on new protective strategies for ovarian function.

Different chemotherapeutic agents cause varying degrees of ovarian damage with diverse mechanisms [3, 5]. Multiple studies have confirmed that alkylating agents are more detrimental to ovarian follicles than other chemotherapeutic agents, and they are most likely to cause premature ovarian failure (POF) [5]. Cyclophosphamide (CTX) is one of the most widely used alkylating agents in the treatment of various cancers and autoimmune diseases, as well as for immunosuppression after blood and marrow transplantations [6]. As the active metabolite of CTX in vivo, phosphoramide mustard induces inter- and intra-strand DNA crosslinks, followed by the generation of double strand breaks (DSBs), the most serious form of DNA damage. Under this circumstance, DNA damage checkpoints are activated to maintain genome integrity with cell cycle arrest and activation of DNA-repair mechanisms, leading to apoptosis and cell death if the DSBs are not repaired [6, 7]. Great efforts have been made to develop new agents with therapeutic potential to preserve ovarian function against chemotherapy through a variety of mechanisms [3, 8–13].

The long-term effects of CTX and other chemotherapeutic agents on female fertility are noted by the reduction of primordial follicles and ovarian hormones, and the increase of follicular atresia, while the short-term effects of CTX on growing follicles is much less concentrated. Granulosa cells (GCs), one of the major groups of cells in the ovaries, are closely involved in hormone synthesis, follicle development and ovulation. GCs in growing follicles are characterized by rapid proliferation, similar to that of cancer cells and they are highly sensitive to chemotherapeutic drugs. CTX was shown to induce GCs apoptosis in growing follicles by activating mitochondria-dependent apoptosis pathway [13]. Thus, how to alleviate the short-term injury before fixing the long-term damage of CTX on GCs is of great interest.

Epigenetic modifications (including DNA/RNA modifications and histone modifications) can cause reversible and heritable changes to specific genes without changing their sequences [14]. Previous studies have found that histone modification enzymes and relevant histone modifications change dynamically in GCs at different developmental stages [15–17]. Histone modifications and chromatin remodeling regulate the expression of several luteinization-related genes, such as steroidogenic acute regulatory protein (*StAR*), cytochrome P450 family 19 subfamily a polypeptide 1 (*Cyp19a1*), and family 11 subfamily a polypeptide 1 (*Cyp11a1*), which in turn regulate hormone synthesis [16, 17], indicating that histone modifications are closely involved in the regulating the functions of GCs. To date, numerous studies have reported on DNA damage and concomitant ovarian damage caused by chemotherapy, but whether epigenetic modifications participate in the regulation of gene expression after chemotherapy has not been widely investigated. Our previous research has shown that the long-term side effects of CTX caused alterations in DNA methylation and histone modifications in oocytes, followed by the transcriptional suppression of multiple maternal genes [18]. Another experimental study also found that maternal exposure to CTX altered the DNA methylation of specific imprinted genes in the oocytes of offspring [19]. These studies indicate that CTX may exert its toxicity not only by directly inducing DNA damage, but also by modifying epigenetic modifications and gene expression.

Tri-methylation of histone H3 lysine 27 (H3K27me3), whose stability is maintained by the balance between the methyltransferases EZH1/2 (enhancer of zeste homolog 1/2), and the lysine-specific demethylase 6A/B (KDM6A/B, also known as UTX/JMJD3), is closely related to transcriptional inhibition and plays an important role in regulating gene expression, balancing cell proliferation and differentiation [20]. In multiple human cancer cells, the inhibition of EZH2 promotes the apoptosis induced by DNA-damaging agents by abrogating both G1 and G2/M checkpoints and cell cycle arrest [21]. However, whether H3K27me3 is involved in CTX-induced ovarian damage, especially in GCs, requires further research.

In this study, we examined the levels of EZH2 protein and H3K27me3 shortly after CTX or its active metabolite 4-hydroperoxy cyclophosphamide (4-HC) treatment to investigate the role of EZH2 and H3K27me3 in CTX-induced GCs apoptosis. Thus, we attempted to elucidate the underlying mechanisms involved in this process and to find future clinical interventions for fertility preservation.

## Results

### Acute exposure to CTX induced DNA damage and apoptosis in GCs of growing follicles

**Fig. 1:**
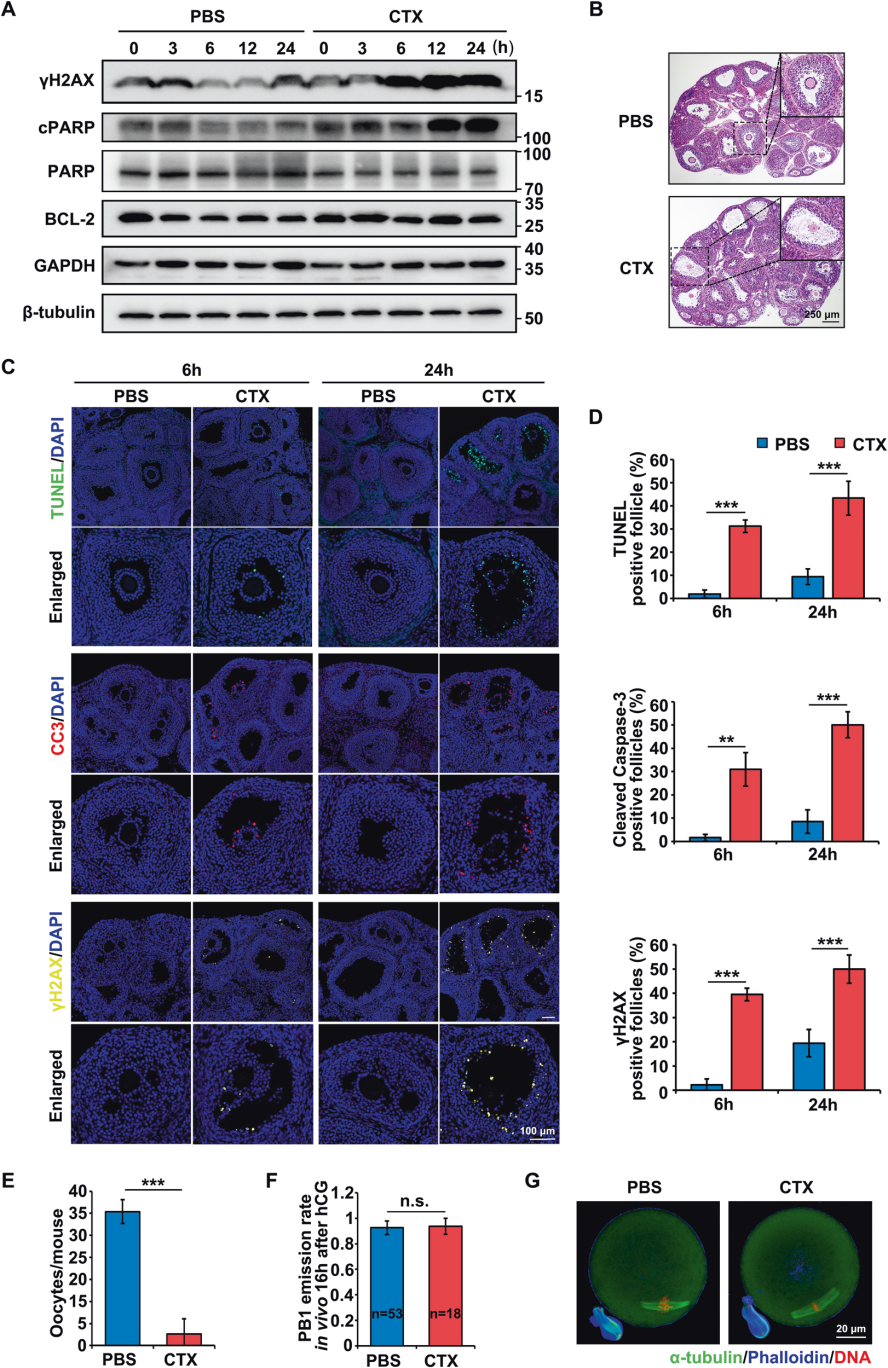
CTX-induced DNA damage and apoptosis in the GCs of growing follicles. **A** Western blot results of γH2AX, cPARP, PARP, and BCL-2 levels of the ovaries from mice treated with or without CTX. GAPDH and β-tubulin were blotted as loading controls. *N* = 6 mice per time point for each group. **B** Hematoxylin and eosin (H&E) staining of ovary sections 24 h after i.p. injection of PBS or CTX. Scale bar, 250 μm. **C** Immunofluorescence staining of ovaries collected at 6 and 24 h after the i.p. injection of PBS or CTX. TUNEL was probed with Alexa Fluor 488 (green). Rabbit monoclonal antibodies CC3 and γH2AX were detected using anti-rabbit IgG (red and yellow, respectively). Cell nuclei were labeled with DAPI (blue). Scale bar, 100 μm; *N* = 6 ovaries from different mice per time point for each group. **D** Quantitative plots for TUNEL, CC3, and γH2AX-positive follicles/total follicles. **E** The number of oocytes collected from the oviducts of the mice with or without CTX treatment after hCG injection. *N* = 8 mice for each group. **F** PB1 emission rates of oocytes collected from the oviducts of mice in vivo 16 h after ovulation induction. The number of analyzed oocytes is indicated (*n*). **G** Immunofluorescent staining for α-tubulin (green), phalloidin (blue), and DNA (red) of the oocytes collected from the oviducts of mice in vivo 16 h after ovulation induction. Scale bar, 20 μm. Data are the mean ± SD of at least three independent experiments. Statistical analyses were carried out using a two-tailed Student’s *t*-test; n.s. non-significant; **P < 0.01; and ***P < 0.001.

To investigate the effects of CTX on the ovaries, 3-week-old ICR females were used to establish animal models, which were similar to adolescent humans and more vulnerable to chemotherapeutics with less aging-related follicular atresia. The DNA damage caused by ionizing radiation or alkylating agents results in the phosphorylation of histone H2AX on serine residue 139 (γH2AX) at the damage sites [22]. If the DSBs are not repaired, further apoptosis would be mediated by cleaved caspase-3 (CC3) [23]. Poly ADP-ribose polymerase (PARP) can be cleaved by caspase-3, resulting in the separation of its N-terminal DNA-binding domain (24 kDa) and C-terminal catalytic domain (also known as cleaved-PARP or cPARP, 89 kDa) [24]. During the late stages of apoptosis, fractured DNA could be labeled and visualized by end-deoxynucleotide transferase catalyzed dUTP end-labeling (TUNEL) reactions. Thus, they can be used as ovarian damage markers. Within 24 h after CTX treatment, there was no significant change in ovary weight (Fig. S1A), but the levels of γH2AX and the ratio of cPARP to PARP protein, as indicators of DNA damage and apoptosis, were evidently increased, especially after 6 h (Fig. 1A). Thus, ovaries collected 6 and 24 h after CTX treatment were selected for further experiments. Compared to the untreated ovaries, the GCs in the rapidly-growing antral follicles after CTX treatment were arranged in a disordered manner with a decreased number of cellular layers morphologically, and there were signs of early apoptotic changes such as nuclear condensation and fragmentation in the ovarian sections under the light microscope (Fig. 1B); this was consistent with the previous research that proliferative cells are more sensitive to CTX [25]. The CTX treatment also caused significant increases in the proportion of TUNEL, γH2AX, and CC3-positive follicles at 6 and 24 h (Fig. 1C, D). However, the proliferative capacity of the surviving GCs was not obviously changed, as indicated by the Ki-67 immunofluorescence (Fig. S1B, C); this could be explained by the short treatment time. Ovulation was activated by the injection of hCG 44 h after a PMSG injection and the ovulated oocytes were collected 16 h after hCG injection. The CTX treatment caused a remarkable decrease in ovulation (Fig. 1E), which was due to the damaged GCs. However, there was no notable influence on the polar body emissions and meiotic spindle configurations of the oocytes (Fig. 1F, G), indicating that ovulation, instead of oocyte meiosis, was seriously impaired after acute exposure to CTX.

The GCs, regulated by the hypothalamic-pituitary-ovarian axis, produce hormones that are crucial for the growth of ovarian follicles and ovulation [26, 27]. This study has thus focused on GCs to help elucidate the mechanism by which CTX impairs ovarian functions and tried to explore new methods for preserving ovarian functions.

### CTX-induced reduction of H3K27me3 and EZH2 associated with GC apoptosis

**Fig. 2:**
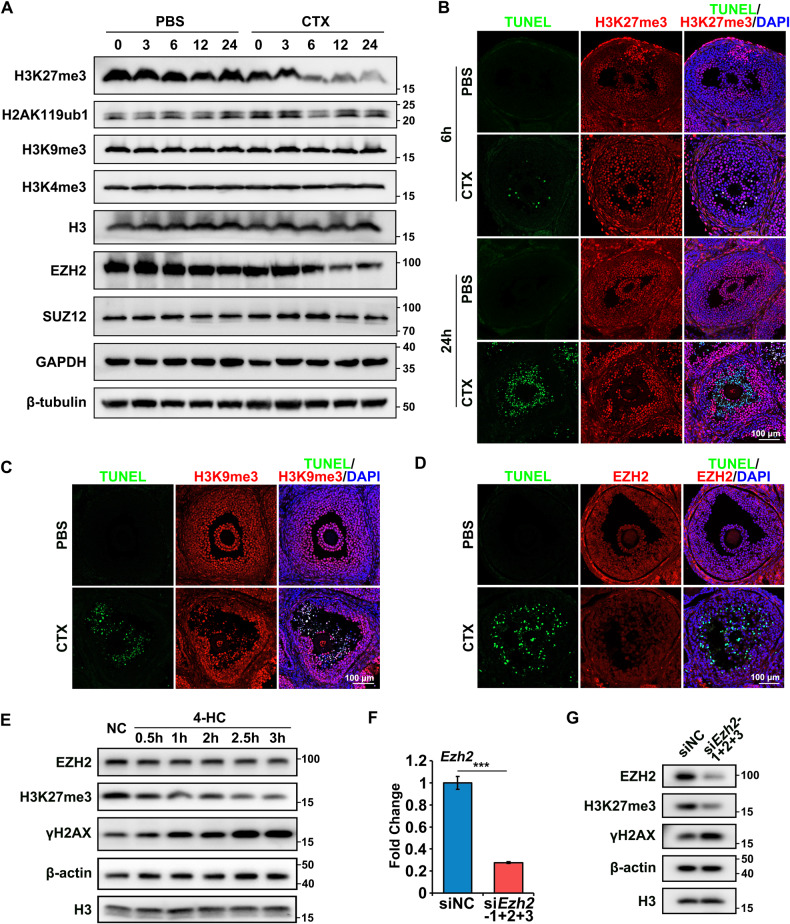
CTX specifically induced the decline of EZH2 and H3K27me3 in GCs. **A** Western blot results of the H3K27me3, H2AK119ub1, H3K9me3, H3K4me3, EZH2, and SUZ12 levels in the PBS and CTX groups. H3, GAPDH, and β-tubulin were blotted as loading controls. *N* = 6 mice per time point for each group. **B**–**D** Immunofluorescence staining of the ovaries collected 6 and 24 h after the i.p. injection of PBS or CTX. Rabbit monoclonal antibodies H3K27me3 (**B**), H3K9me3 (**C**), and EZH2 (**D**) were detected using anti-rabbit IgG (red). TUNEL was probed with Alexa Fluor 488 (green). Cell nuclei were labeled with DAPI (blue). Scale bar, 100 μm. *N* = 6 ovaries from different mice per time point for each group. **E** Western blot results for EZH2, H3K27me3, and γH2AX levels in the negative control (NC) and 4-HC treatment groups. β-actin and H3 were blotted as loading controls. **F** Quantitative expression of *Ezh2* in the siNC and si*Ezh2*-1 + 2 + 3 groups. **G** Western blot results of EZH2, H3K27me3, and γH2AX levels in the siNC and si*Ezh2*-1 + 2 + 3 groups. β-actin and H3 were blotted as loading controls. Data are the mean ± SD of at least three independent experiments. Statistical analyses were carried out using a two-tailed Student’s *t*-test; ***P < 0.001.

The H3K27me3 was found to decrease gradually after CTX treatment and no significant changes were detected in the other histone modifications, such as mono-ubiquitination of histone H2A lysine 119 (H2AK119ub1), tri-methylation of histone H3 lysine 4 (H3K4me3), and tri-methylation of histone H3 lysine 9 (H3K9me3) (Fig. 2A). Further immunofluorescence experiments showed that the fluorescence signals of H3K27me3 were dramatically decreased in apoptotic GCs, as was indicated by the TUNEL staining (Fig. 2B). Meanwhile, the fluorescence signals of H3K9me3 in apoptotic GCs were not altered (Fig. 2C), which further confirmed that the H3K27me3 levels were specifically decreased in the GCs after CTX treatment. Therefore, the influence of CTX on the histone methyltransferase responsible for H3K27me3 was investigated further. The results identified that EZH2, which was the catalytic core of the polycomb repressive complex 2 (PRC2) to generate H3K27me3, also decreased gradually after CTX treatment, which was similar to the changes in H3K27me3 (Fig. 2A, D), while another PRC2 component SUZ12 showed no significant changes (Fig. 2A). Overall, these results suggested that the decrease of H3K27me3 may result from EZH2 reduction after CTX treatment.

To further verify the influence of CTX on H3K27me3 and EZH2, an active metabolite form of CTX 4-HC [28] was applied to primary GCs in vitro. H3K27me3 and EZH2 were decreased in primary GCs with the extending 4-HC incubation time, while the γH2AX levels were evidently increased (Fig. 2E), resembling the pharmacological effects of the CTX treatment in vivo. To confirm whether the level of H3K27me3 was related to apoptosis, siRNAs were used to knock down the expression of *Ezh2* in primary GCs. The expression of *Ezh2* mRNA decreased by approximately 70% (Fig. 2F) and the EZH2 protein decreased (Fig. 2G) after application of the si*Ezh2* for 48 h. A knockdown of *Ezh2* led to decreased H3K27me3 and stimulated apoptosis as the γH2AX level was evidently increased (Fig. 2G), which was consistent with the in vivo results. Overall, the CTX treatment reduced the level of EZH2 and H3K27me3, which were closely related to apoptosis in GCs.

### Transcriptional overactivation of transcription- and apoptosis-related genes induced by CTX

**Fig. 3:**
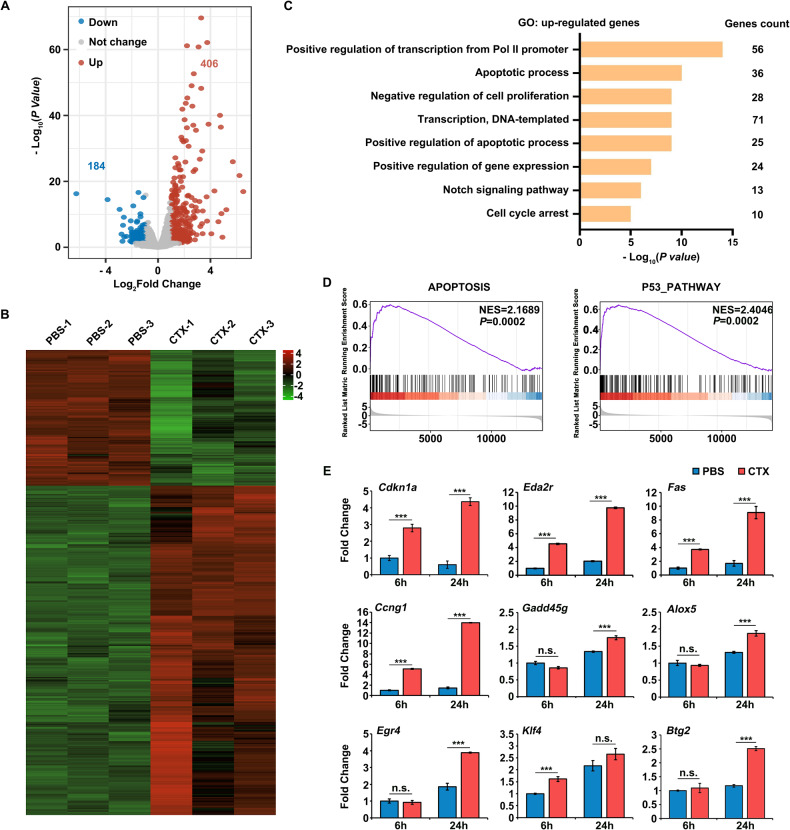
RNA sequencing analyses of GCs isolated from the ovaries of mice treated with or without CTX for 6 h. **A** Volcano plot comparing the transcripts of the GCs isolated from the ovaries of mice treated with or without CTX for 6 h. Transcripts that increased or decreased by more than 2-fold in the GCs isolated from the ovaries of mice treated with CTX were highlighted in red or blue, respectively. GCs isolated from 3 mice were pooled. *N* = 3 biological replicates. **B** Heatmap of differentially expressed genes in the GCs isolated from the ovaries of mice treated with or without CTX for 6 h sorted by adjusted *P* value by plotting their log_2_ transformed expression values in replicates. **C,** **D** GO (**C**) and GSEA (**D**) analysis of significantly enriched pathways in the upregulated gene sets of GCs isolated from the ovaries of mice treated with CTX. **E** Relative expression of cyclin-dependent kinase inhibitor 1A (*Cdkn1a*, also known as *p21*), ectodysplasin A2 receptor (*Eda2r*), TNF receptor superfamily member 6/Fas cell surface death receptor (*Fas*), cyclin G1 (*Ccng1*), growth arrest and DNA-damage-inducible 45 gamma (*Gadd45g*), arachidonate 5-lipoxygenase (*Alox5*), early growth response 4 (*Egr4*), Kruppel-like transcription factor 4 (*Klf4*), and BTG anti-proliferation factor 2 (*Btg2*) mRNA levels by RT-qPCR in the GCs isolated from the ovaries of mice treated with or without CTX. *N* = 6 mice per time point for each group. Data are the mean ± SD of at least three independent experiments. Statistical analyses were carried out using two-tailed Student’s *t*-test; n.s. non-significant; ***P < 0.001.

Given that the apoptosis of CTX-treated GCs was possibly related to alternations in the gene expression and signaling pathways, RNA-seq analyses of the GCs from the CTX-treated and control groups (*N* = 3, respectively) were performed 6 h after CTX treatment. The global transcriptional changes across the groups (PBS *v.s*. CTX) were presented using a volcano plot and hierarchical clustering. In total, 590 differentially expressed genes (DEGs) were identified, of which 406 (68.81%) were upregulated and 184 (31.19%) were downregulated after CTX treatment (Fig. 3A, B). Consistent with the previously verified CTX-induced reduction of H3K27me3, the epigenetic modifications associated with inactive transcription, the CTX treatment was more effective at upregulating gene expression. Gene ontology (GO) analysis of the genes that were upregulated by CTX treatment indicated that these genes were mainly related to transcriptional regulation, apoptosis, negative regulation of cell proliferation, and some other pathways (Fig. 3C). Gene set enrichment analysis (GSEA) also indicated that the upregulated genes were closely related to apoptosis and the p53 pathway (Fig. 3D). Further RT-qPCR was conducted to confirm the CTX-induced upregulation of these genes. A number of the upregulated genes were involved in pathways such as apoptosis (*Cdkn1a*, *Eda2r*, and *Fas*), DNA damage (*Ccng1* and *Gadd45g*), and transcription (*Egr4*, *Klf4*, and *Btg2*). Furthermore, the increase in these genes was more apparent with time (Fig. 3E), which may account for the increased apoptosis and altered gene expression in the GCs after CTX treatment.

### Reduced H3K27me3 peaks on upregulated genes induced by CTX treatment

**Fig. 4:**
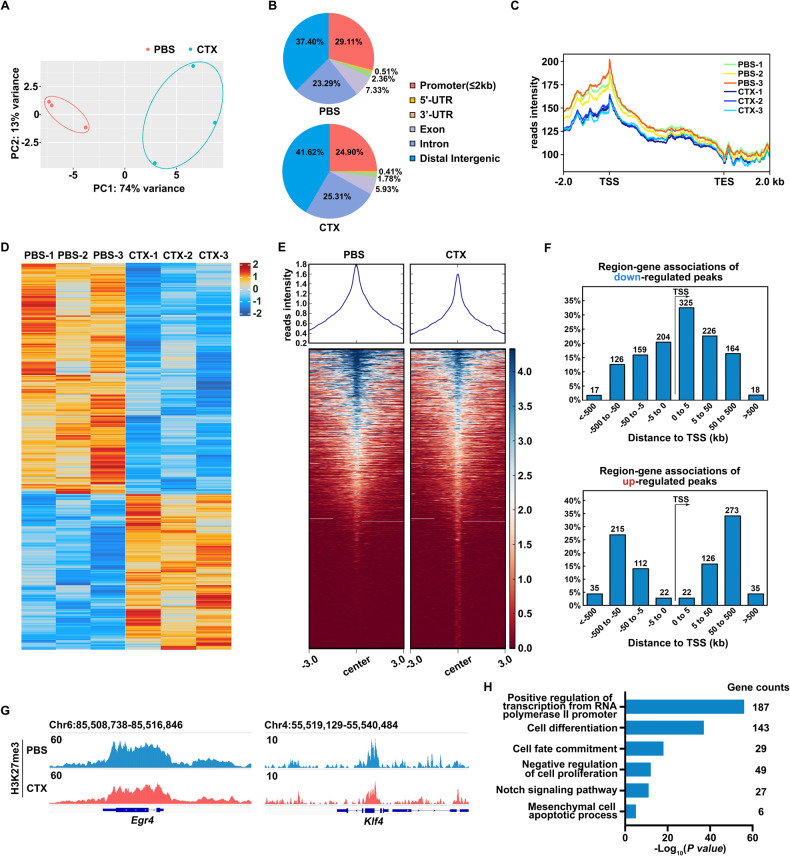
Anti-H3K27me3 CUT&Tag assay of GCs isolated from the ovaries of mice treated with or without CTX for 6 h. **A** PCA analysis of the targeted H3K27me3 peaks of the GCs isolated from the ovaries of mice treated with or without CTX for 6 h. GCs isolated from 3 mice were pooled. *N* = 3 biological replicates. **B** Distribution of H3K27me3 peaks on the functional regions of genes. **C** Metaplot showing H3K27me3 enrichment with 2 kb upstream and downstream of the gene body in the GCs isolated from the ovaries of mice treated with or without CTX. **D** Heatmap showing the genes with significantly low or high H3K27me3 peaks in the GCs isolated from the ovaries of mice treated with or without CTX. **E** Metaplot and heatmap showing H3K27me3 enrichment of individual genes in GCs isolated from the ovaries of mice treated with or without CTX. **F** Distribution of significantly downregulated (top) and upregulated (bottom) H3K27me3 peaks relative to the TSS sites. **G** Genome browser view showing H3K27me3 enrichment near *Egr4* and *Klf4* in GCs isolated from the ovaries of mice treated with or without CTX. **H** GO analysis of significantly enriched pathways in the gene sets mapped to the downregulated H3K27me3 peaks in GCs isolated from the ovaries of mice treated with CTX.

To determine whether the upregulation of selected genes induced by CTX treatment were associated with loss of H3K27me3 on specific gene regions, an anti-H3K27me3 CUT&Tag experiment was performed [29] using GCs under the same condition with the RNA-seq experiments. Principal-component analysis (PCA) on targeted H3K27me3 peaks showed that the CTX-treated groups were readily separated from the control groups with a high consistency in each group (Fig. 4A). The proportion of H3K27me3 peaks in the promoter region showed the most obvious reduction in the GCs after CTX treatment (29.11% *v.s*. 24.90%) (Fig. 4B, C).

Of the 1650 genes with noticeable altered H3K27me3 peaks after CTX treatment, 971 (58.84%) had lower and 679 (41.16%) had higher H3K27me3 peaks (Fig. 4D). In both groups, the H3K27me3 peaks were mainly distributed around the transcriptional start site (TSS) (Fig. 4C, E), indicating its function of regulating gene expression, as reported in previous studies [20]. As the reduction of H3K27me3 has been previously confirmed (Fig. 2A), overall H3K27me3 peaks were lower than that of the control group after CTX treatment (Fig. 4C, E). Those regions with downregulated H3K27me3 peaks were also enriched around the TSS (Fig. 4F, top), and those regions with upregulated H3K27me3 peaks excluded TSS (Fig. 4F, bottom), suggesting that downregulated H3K27me3 around the promoters participated in CTX-induced gene regulation.

Among the H3K27me3-marked genes activated by CTX treatment, *Egr4* and *Klf4* were both transcription factors extensively studied in cancers. Early growth response protein 4 (*Egr4*), a transcription factor belonging to the EGR family of zinc-finger transcription factors, is reportedly involved in the regulation of cell growth and apoptosis in cancers [30, 31]. Krüppel-like factor 4 (*Klf4*), a member of the evolutionarily conserved family of zinc-finger transcription factors, is a transcription factor regulating cell proliferation, differentiation, and apoptosis [32]. In this study, abnormal expression of *Egr4* and *Klf4* might lead to further aberrant expression of downstream apoptosis genes in the GCs. Consistent with the previously verified upregulation of *Egr4* and *Klf4* transcription after CTX treatment (Fig. 3E), the H3K27me3 peaks were remarkably lower on their promoters, which demonstrated that the upregulation of these genes directly resulted from the reduced H3K27me3 levels (Fig. 4G). Besides, GO analysis showed that the genes with reduced H3K27me3 peaks after CTX treatment were mainly related to pathways about transcriptional regulation, cell differentiation, and negative regulation of cell proliferation (Fig. 4H), which was consistent with the pathways in which upregulated genes were enriched from the RNA-seq data (Fig. 3C).

Taken together, these results suggest that the reduction of H3K27me3 peaks in the promoter region induced by CTX was directly and indirectly involved in the abnormal gene expression and following apoptosis of GCs.

### GSK-J4 relieved CTX-induced apoptosis and transcription aberrance

**Fig. 5:**
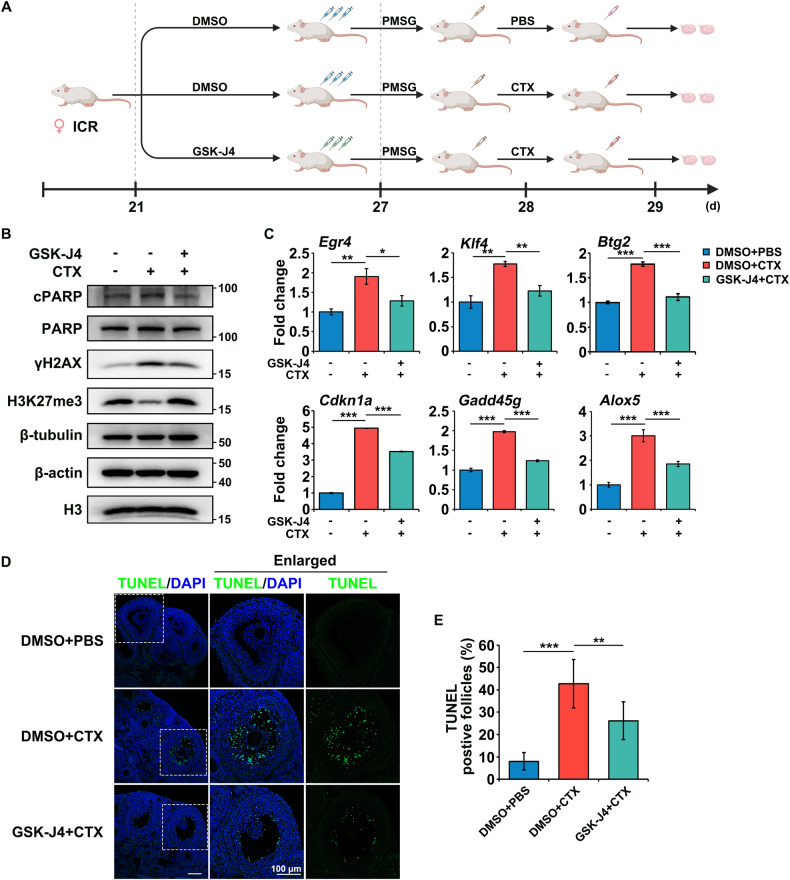
Protective potential of GSK-J4 against CTX-induced GCs apoptosis and transcription abnormality. **A** Schematic diagram showing the grouping and the dosing schema of 3-week-old female ICR mice. GSK-J4 or DMSO was i.p. injected three times in a week (days 21, 23, and 25, as bounded by the dashed lines). PMSG were i.p. injected once at day 27. CTX or PBS was i.p. injected once 22 h after PMSG injection (day 28). The ovaries were harvested 24 h after CTX injection (day 29). **B** Western blot results showing the cPARP, PARP, γH2AX, and H3K27me3 levels in the PBS and CTX groups. β-tubulin, β-actin, and H3 were blotted as loading controls. *N* = 4 mice for each group. **C** Relative expression of *Egr4*, *Klf4*, *Btg2, Cdkn1a*, *Gadd45g*, and *Alox5* mRNA levels by RT-qPCR in the GCs isolated from the ovaries of mice. *N* = 4 mice for each group. **D** Immunofluorescence staining of the ovaries collected from the above three groups. TUNEL was probed with Alexa Fluor 488 (green). Cell nuclei were labeled with DAPI (blue). Scale bar, 100 μm. *N* = 6 ovaries from different mice for each group. **E** Quantitative plots of TUNEL-positive follicles/total follicles. Data are the mean ± SD of at least three independent experiments. Statistical analyses were carried out using two-tailed Student’s *t*-test; *P < 0.05; **P < 0.01; and ***P < 0.001.

To explore methods to rescue ovarian functions from CTX toxicity, we managed to alleviate H3K27me3 reduction using a GSK-J4 pre-treatment (Fig. 5A), which was a potent dual inhibitor of H3K27me2/3-demethylases KDM6A/B. Administration of GSK-J4 (5 mg/kg, three times a week) before CTX treatment could attenuate the CTX-induced reduction of H3K27me3 effectively (Fig. 5B). The GSK-J4 pre-treatment reduced the level of γH2AX and the ratio of cPARP to PARP protein (Fig. 5B), indicating its protective effects against CTX. The expression levels of the genes upregulated after CTX treatment (such as *Klf4*, *Egr4*, *Btg2*, *Cdkn1a*, *Gadd45g,* and *Alox5*) were also partially restored (Fig. 5C), suggesting that GSK-J4 could rescue the CTX-induced abnormal overexpression of specific genes. The apoptotic index (TUNEL-positive follicles/total follicles) was also rescued in the GSK-J4-pretreated ovaries when compared with the DMSO-treated ovaries after CTX treatment, suggesting the inhibition of CTX-induced apoptosis (Fig. 5D, E). Taken together, GSK-J4 was found to restore H3K27me3 levels and alleviate the GCs apoptosis induced by CTX. Thus, the abnormal gene expression and accompanying GCs apoptosis induced by CTX could be reversed by restoring H3K27me3 levels with GSK-J4.

## Discussion

**Fig. 6:**
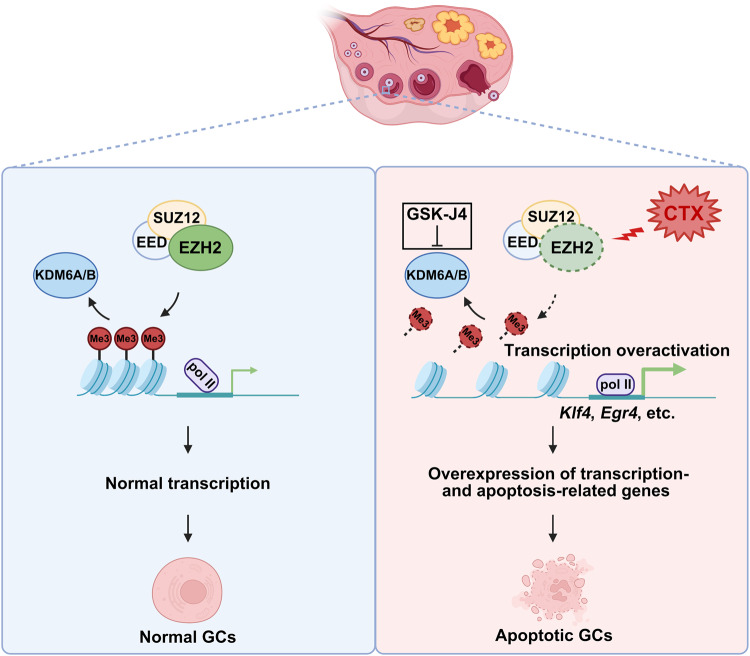
Schematic diagram showing how the EZH2-H3K27me3 axis modulates CTX-induced transcription aberrance and apoptosis in the ovarian GCs of growing follicles. Under normal conditions, a stable H3K27me3 status balanced by H3K27 methyltransferases and demethylases maintains appropriate gene expression and cellular homeostasis in GCs. Acute exposure to CTX induces rapid decline of EZH2, the catalytic component of PRC2 complex, resulting in the loss of H3K27me3 on the promoters of transcription-related genes, led to further aberrant transcription of apoptosis-related genes and cell apoptosis in GCs. A pre-treatment with a KDM6A/B dual inhibitor GSK-J4 can rescue H3K27me3 status, alleviate gene overexpression, DNA damage, and apoptosis after the CTX treatment.

CTX is widely used as a chemotherapeutic and immunosuppressive drug for the treatment of multiple tumors and autoimmune diseases. However, CTX is known to have a negative impact on fertility. Our study has shown that after CTX treatment, the GCs in the growing follicles exhibited a rapid decline of H3K27me3, an epigenetic modification closely related to transcriptional inhibition. Mechanistically, CTX reduced the EZH2 protein level, which is an H3K27 methyltransferase, resulting in the loss of H3K27me3 on specific gene promoters, and aberrant expression of transcription- and apoptosis-regulative genes, which further amplified CTX toxicity. To rescue the H3K27me3 loss induced by CTX, a pre-treatment with a KDM6A/B dual inhibitor GSK-J4 was adopted to maintain the H3K27me3 status. This approach restored the H3K27me3 level in GCs and alleviated CTX-induced gene overexpression, DNA damage, and apoptosis in vivo (Fig. 6).

A chemotherapy-induced ovarian failure mouse model was generated using i.p. injection of CTX. Consistent with previous studies, CTX caused a significant increase in GCs apoptosis morphologically [8–13], while the ovarian weight showed no change within 24 h after CTX treatment (Fig. S1A), and this is likely to be associated with the short treatment time. The results of this study showed that the γH2AX, cPARP/PARP, and CC3 levels as well as the TUNEL signals in the ovaries from the CTX group, were evidently increased, especially in the GCs (Fig. 1A, C, D). Further investigation into the epigenetic modifications showed that H3K27me3 and its methyltransferase EZH2 decreased after CTX treatment in a time-dependent manner in vivo (Fig. 2A, B, D). The application of 4-HC and si*Ezh2* also had identical effects on the primary GCs in vitro with increased DNA damage, and decreased levels of H3K27me3 and EZH2 (Fig. 2E–G), indicating that CTX specifically induced the decline of EZH2 and H3K27me3, which in turn regulated GC apoptosis. The reduction of EZH2 may result from ataxia telangiectasia mutated kinase-mediated phosphorylation of EZH2 under genotoxic stress to decrease its stability [22, 33, 34], but this will require further investigation. The correlation between EZH2 and apoptosis is supported by studies in which microRNA 26a targets *Ezh2* to reduce EZH2 expression, inducing GCs apoptosis in mice [35] or colorectal cancer cell apoptosis [36]. While in renal tubular epithelial cells, similar to our findings, EZH2 inhibition with 3-deazaneplanocin A (DZNep) has been found to upregulate *Deptor* by reducing H3K27me3 in the promoter region, which subsequently inhibits mTORC1/2 activities, downregulates the expression of apoptosis suppressor genes, and results in cell apoptosis [37].

GCs are the main secretors of ovarian hormones including estrogen and progesterone. The function of GCs is not solely related to oocyte growth, maturation and ovulation, but also involved in subsequent fertilization and early embryo development [26, 27, 38]. In this study, ovulation was severely damaged within 24 h of CTX treatment (Fig. 1E), which was in line with increased GCs apoptosis. Previous studies on ovarian injury induced by CTX have predominantly focused on the mitochondrial oxidative stress-related apoptosis in GCs, but the abnormality of gene expression profiles and the underlying mechanisms are largely unknown. Thus, RNA-seq analyses were performed using the GCs collected 6 h after CTX treatment and from the control group collected at the same time point. With 406 (68.81%) upregulated genes and 184 (31.19%) downregulated genes (Fig. 3A, B), the CTX treatment tended to activate the gene transcription of the GCs, as pathways related to positive regulation of transcription were also enriched by the GO analysis (Fig. 3C). Consistent with the apoptosis phenotype, CTX induced the upregulation of apoptosis-related genes, and this was confirmed by the GO analysis, GSEA analysis and RT-qPCR (Fig. 3C–E). Importantly, the upregulation of transcription-related genes may result in larger cascade effects on aberrant gene transcription. In this study, the upregulation of *Klf4* was induced by the depletion of EZH2 and H3K27me3. A previous study has reported that *Klf4* negatively regulates the long noncoding RNA PiHL and impairs PiHL’s function to remove EZH2 from the promoter region, which in turn inactivates the transcription of a high mobility group AT-hook 2 (*Hmga2)* via EZH2-mediated H3K27 methylation. The downregulation of *Hmga2* could sensitize colorectal cancer cells to oxaliplatin and promote drug-induced cell apoptosis by inactivating the PI3K/AKT pathway [39]. This regulatory axis may be involved in negative feedback to help mitigate the reduction of EZH2 and H3K27me3 induced by CTX but may also contribute to the amplification of aberrant gene transcription and cell apoptosis after CTX treatment. Future studies will be needed to further investigate this regulatory mechanism.

Multiple studies have shown that changes in the gene expression of GCs could be regulated by epigenetic modifications, including DNA methylation and histone modifications, except for the traditional activation of transcription factors. The variability of DNA methylation in mural GCs from the diminished ovarian reserve (DOR) group is significantly elevated when compared with normal groups [40]. In addition, the expression of the fragile X messenger ribonucleoprotein 1 (*FMR1*) gene in the GCs of DOR patients is approximately 2-fold higher than that of the control group, resulting from epigenetic changes in the *FMR1* gene including H3K9ac, H3K9me2, and H3K9me3 [41]. Sen et al. defined the role of androgen in regulating the expression of key ovarian genes through the modulation of H3K27me3 by inhibiting the expression and activity of EZH2 and inducing the expression of KDM6B in GCs [42, 43]. In rats, H3K4me3 increases, while H3K9me3 and H3K27me3 decreases in the promoter regions of the *StAR* and *Cyp11a1* gene after ovulation induction, contributing to the rapid induction of *StAR* and *Cyp11a1*, both of which are involved in the synthesis of progesterone [16, 17]. Another study also reported that H3K9me3 and H3K27me3 decrease in the C/EBPβ binding region of the *Vegf* gene in GCs undergoing luteinization after hCG injection [44]. In the meantime, histone H3/H4 acetylation and H3K4me3 decrease, and H3K27me3 increases in the *Cyp19a1* promoter after ovulation induction, contributing to the rapid suppression of *Cyp19a1*, a key aromatase for estrogen synthesis [17]. Progressive decreases in DNA methylation in approximately 40% of genes [45] and the variable expression of histone modification enzymes, including *Ezh2*, *Setdb2*, *Hdac4*, *Hdac10*, and *CIIta* can be detected in GCs undergoing luteinization after ovulation induction [15]. In porcine, H3K27me3 transcriptionally represses the transcription factor runt-related transcription factor 1 (*Runx1*), which in turn influences GCs’ steroidogenesis, anti-apoptotic activity, and cell proliferation activity [46]. A study using GC-specific *Kdm6b*-KO mice has demonstrated that KDM6B promotes follicle growth by regulating the expression of genes critical for mitochondrial function in GCs [47]. The above studies identify the importance of H3K27me3 stability in the regulation of GCs’ function and cell viability. However, few studies have addressed the role of H3K27me3 during GCs apoptosis induced by CTX.

To further discover whether changes in GCs gene expression after CTX treatment are regulated by H3K27me3, an anti-H3K27me3 CUT&Tag experiment was conducted. The distribution of the H3K27me3 peaks showed an evident reduction around the promoters (Fig. 4B, C). As H3K27me3 is a transcriptional repressive marker, the removal of H3K27me3 around the promoters de-condenses the chromatin and enhances transcription factor accessibility to promote the transcription of certain genes. In this study, the abundance of H3K27me3 peaks around the TSS were substantially lower than that of the control group (Fig. 4C, E, F). Genes upregulated in GCs after CTX treatment showed reduced H3K27me3 peaks (Fig. 4G) in accordance with transcriptional activation in the CTX group found in our RNA-seq data. The results have defined the role of H3K27me3 in CTX-induced apoptosis and the abnormal changes of gene expression in GCs.

Chromatin remodeling induced by the loss of H3K27me3 might enhance CTX toxicity of the GCs in this study. Previous studies have confirmed that compacted chromatin exhibit greater resistance to DNA damage [48, 49]. A recent study on AML cells has confirmed that the inhibition of EZH2 to de-condense the chromatin could enhance the chemotherapeutic accessibility to chromatin, chemotherapy-induced DNA damage, and cell apoptosis [50]. In this study, restoring the H3K27me3 status by inhibition of the H3K27 demethylases KDM6A/B might exert its protective potential by condensing the chromatin, decreasing DSBs formation, and partially resisting CTX-induced DNA damage and subsequent apoptosis in GCs (Fig. 5).

In conclusion, this is the first study to compare the global gene expression profiles between the GCs with and without the acute CTX treatment. We elucidate the role of EZH2-H3K27me3 axis in gene regulation of CTX-treated GCs. Mechanistically, CTX induces a decline in H3K27me3 via a reduction in EZH2. Loss of H3K27me3 on promoters activates the expression of transcription-related and downstream apoptosis genes, which amplifies the aberrance of downstream gene expression and GCs apoptosis (Fig. 6). A thorough understanding of this mechanism by which acute exposure of CTX damage ovarian function could contribute to new fertility protection and preservation strategies for female patients with cancer.

## Materials and methods

### Animal models

Female ICR mice were obtained from the Animal Center of Sir Run Run Shaw Hospital (Hangzhou, Zhejiang Province, China) and housed in specific pathogen-free conditions with temperature (22 ± 1 °C) and humidity (60 ± 10%) controls on a 12 h light/12 h dark cycle, with ad libitum access to water and regular rodent chow. All the animal protocols were approved by Zhejiang University Animal Care and Use Committee (Hangzhou, Zhejiang Province, China). 3-week-old female mice were intraperitoneal (i.p.) injected with 5 IU of pregnant mare’s serum gonadotropin (PMSG) to activate follicular development. For CTX-induced ovarian damage models, the mice were randomly divided into two groups: PBS and CTX (*N* = 6 per group) 22 h after PMSG injection. The mice were weighed and then treated with a single i.p. injection of CTX (120 mg/kg, Baxter, Milan, Italy), or an equal volume of phosphate buffer saline (PBS) as the control. Both groups were sacrificed immediately or 3, 6, 12, or 24 h after CTX treatment, and ovaries were collected for further analysis. For the GSK-J4-pretreated models (*N* = 4 per group), GSK-J4 (HY-15648B; MedChemExpress, Monmouth Junction, NJ, USA) and dimethyl sulfoxide (DMSO) as a solvent control were i.p. injected into 3-week-old female mice three times in a week before PMSG and CTX i.p. injection (Fig. 5A).

### Ovulation induction and oocyte collection

Mice were injected with 5 IU of human chorionic gonadotropin (hCG) 44 h after the PMSG injection to induce ovulation and they were sacrificed 16 h later. Oocytes and cumulus complexes were harvested from the oviducts of the mice in the M2 medium (M7167; Sigma-Aldrich, St. Louis, MO, USA). The numbers of ovulated oocytes and the first polar body (PB1) emission rates were analyzed after digestion with hyaluronidase.

### Immunofluorescence

For the immunofluorescence of oocytes, oocytes were fixed in 4% paraformaldehyde in PBS for 30 min, then permeabilized in PBS containing 0.5% Triton X-100 for 20 min and blocked with 1% bovine serum albumin (BSA) in PBS for 30 min sequentially. After being incubated with the primary antibodies for 1 h at 26 ± 1 °C, the oocytes were labeled with Alexa Fluor 568- or 488-conjugated secondary antibodies and 4′,6-diamidino-2-phenylindole (DAPI) for 30 min.

For the immunofluorescence of the ovary section, ovaries were collected, fixed in 4% paraformaldehyde in PBS for at least 16 h, and dehydrated using a 10%, 20%, and 30% sucrose gradient until the ovaries completely sank to the bottom of the centrifuge tube. The ovaries were embedded in optimum cutting temperature (O.C.T., Sakura, USA) compound and frozen in liquid nitrogen. Frozen ovary sections that were 10 μm were made with a microtome (Leica, Weztlar, Germany) and stored at −80 °C. When the immunofluorescence staining was performed, the sections were placed at 26 ± 1 °C for 5 min, rinsed 3 times in PBS for 5 min each time, permeabilized and blocked in PBS containing 0.3% Triton X-100 and 5% BSA for 1 h sequentially. After being incubated with the primary antibodies for at least 16 h, the sections were rinsed 3 times in PBS for 5 min each time, and then labeled with Alexa Fluor 568- or 488-conjugated secondary antibodies and DAPI for 30 min. Imaging was performed on a LSM710 confocal microscope (Zeiss, Oberkochen, BW, Germany). The antibodies used are listed in Supplementary Table 1.

### Western blot analysis

Western blot analysis was conducted in accordance with a previous study [18]. Briefly, proteins were extracted from the ovaries (after being weighed) or primary GCs with RIPA lysis buffer (R0010; Solarbio, Beijing, China) containing a protease inhibitor cocktail (P8340; Sigma-Aldrich, St. Louis, MO, USA) and diluted with Laemmli protein sample buffer (1610747; Bio-Rad, Hercules, CA, USA). After denaturation for 10 min at 95 °C, equal amounts of extracted proteins (approximately 10 µg each) were separated via electrophoresis on 10% sodium dodecyl sulfate-polyacrylamide gel (SDS-PAGE) and transferred to polyvinylidene difluoride (PVDF) membranes (Millipore, Billerica, MA, USA). The membranes were blocked in TBST buffer containing 5% skimmed milk for 1 h and then incubated with primary antibodies for at least 16 h at 4 °C. The antibodies used are listed in Supplementary Table 1. The membranes were rinsed 3 times for 5 min each time with TBST buffer, then incubated with secondary antibodies and visualized using enhanced chemiluminescence (WBKLS0500; Millipore, Billerica, MA, USA) with a ChemiDoc Touch imaging system (Bio-Rad, Hercules, CA, USA).

### Primary GCs in vitro culture and treatment

Ovaries were collected from 3-week-old mice 24 h after i.p. injection of the 5 IU PMSG to harvest the GCs, as described previously [51]. Antral follicles were punctured with a 26.5-G needle to release the GCs. The GCs were then cultured with DMEM/F12 (Invitrogen, Carlsbad, CA, USA) supplemented with 5% (v/v) fetal bovine serum (Gibco, Carlsbad, CA, USA) and 1% (v/v) penicillin and streptomycin (Meilunbio, Dalian, China) in 12-well plates. To evaluate the direct role of CTX, the GCs were treated with 2.5 μM 4-HC (the in vitro active form of CTX, HY-117433; MedChemExpress, Monmouth Junction, NJ, USA).

### Small interfering RNA transfection

To determine the role of EZH2 and H3K27me3 in the GCs, the GCs were transfected with small interfering RNAs (siRNAs) targeting *Ezh2* (si*Ezh2*) or a negative control siRNA (siNC) for 48 h using the Lipofectamine™ 3000 Transfection Reagent (L3000015; Thermo Fisher Scientific, Waltham, MA, USA), in accordance with the manufacturer’s instructions. These siRNAs were purchased from RiboBio Co., Ltd (Guangzhou, China). The si*Ezh2*-1, si*Ezh2*-2, si*Ezh*2-3 sequences were 5′-GCTGATGAAGTAAAGACTA -3′, 5′- GGATAATCGAGATGATAAA -3′ and 5′- CAGAGAATGTGGATTTATA -3′, respectively.

### RNA-seq library preparation and gene expression analysis

RNA-seq was performed to compare the global gene expression profiles between the GCs with CTX treatment and the GCs without CTX treatment (*N* = 3 biological replicates each group). Samples (GCs extracted from ovaries of 3 mice per sample) were collected from mice 6 h after treatment with or without CTX for RNA-seq. High throughput sequencing and bioinformatics analyses were conducted at Novogene (Beijing, China). A volcano plot of the differentially expressed genes (DEGs) was generated using the gplots package in Bioconductor. DEGs with a log_2_(CTX/PBS) > 1 were labeled in red (*P* < 0.05); DEGs with log_2_(CTX/PBS) < − 1 were labeled in blue (*P* < 0.05). Only transcripts with more than a two-fold change and a corrected *P* < 0.05 were considered statistically significant.

### CUT&Tag library preparation, sequencing, and analysis

GCs samples were collected under the same condition as for the RNA-seq experiments and in accordance with the manufacturer’s instructions (Novogene, Beijing, China). Library construction was performed as described previously [29]. Briefly, the GCs samples were bound to Concanavalin A-coated magnetic beads, and the cell membrane was permeabilized using Digitonin. The enzyme pA-Tn5 transposase precisely binds the DNA sequence near the target protein under the antibodies guidance and results in a factor-targeted tagmentation. DNA sequences were then tagmented, with adapters added at the same time to both ends, which could be enriched by PCR to form the sequencing-ready libraries. After the PCR reaction, libraries were purified with the AMPure beads and library quality was assessed on the Agilent Bioanalyzer 2100 system. The clustering of the index-coded samples was performed on a cBot Cluster Generation System using a TruSeq PE Cluster Kit v3-cBot-HS (Illumina, San Diego, CA, USA), according to the manufacturer’s instructions. The library preparations were sequenced on Illumina Novaseq platform. The CUT&Tag reads were aligned to the mouse genome mm10 using BWA (Version 0.7.12). Only uniquely mapped (MAPQ ≥ 13) and de-duplicated reads were used for further analysis. All peak calling was performed with MACS2 (Version 2.1.0). By default, peaks with a q-value threshold of 0.05 were used for all data sets. ChIPseeker was used to retrieve the nearest genes around the peak and annotate the genomic region of the peak. Peak-related genes can be confirmed using the ChIPseeker, and this was followed by Gene Ontology (GO) enrichment analysis to identify the functional enrichment results. GO enrichment analysis was implemented using the GOseq R package, in which gene length bias was corrected. GO terms with corrected *P* < 0.05 were considered significantly enriched by peak-related genes. Peaks of different groups were merged using the ‘bedtools merge’. The mean RPM for each group was calculated in the merge peak. Only peaks with more than two-fold changes in RPM were considered as differential peaks. Genes associated with different peaks were identified using ChIPseeker.

### Real-Time quantitative PCR (RT-qPCR)

RNA was extracted from the GCs collected as described above using the RNeasy Mini Kit (74104; Qiagen, Hilden, NRW, Germany), in accordance with the manufacturer’s protocol. Reverse transcription was conducted using HiScript II Reverse Transcriptase (R201-1; Vazyme, Nanjing, China). RT-qPCR was performed using the SYBR qPCR Master Mix (Q511-02; Vazyme, Nanjing, China) on a CFX96 Real-time System (Bio-Rad, Hercules, CA, USA). The relative mRNA expression levels were normalized to the endogenous *Actb* mRNA levels and compared with those in the control group, and all RT-qPCR assays were performed in triplicate. The primers are shown in Supplementary Table 2.

### Statistical analysis

GraphPad 9.0 and SPSS 26.0 software were used for all statistical analyses. Definition of “center values” as mean; definition of error bars as SD. All quantitative results were shown as the mean ± SD. Each measurement was performed using the data from at least three independent experiments. Differences between two groups were evaluated using a two-tailed unpaired Student’s *t*-tests. *P* < 0.05 indicated statistical significance.

### Supplementary information

Supplementary Figures and Tables

Uncropped western blot

Reproducibility checklist

## Data Availability

All data are included in this published article and its supplementary information file. RNA-seq and CUT&Tag data have been deposited in the NCBI Gene Expression Omnibus database (GSE235907 and GSE236296, respectively).
