# Supplementary material for: The EZH2-H3K27me3 axis modulates aberrant transcription and apoptosis in cyclophosphamide-induced ovarian granulosa cell injury
Source: Cell Death Discov. 2023 Nov 14;9:413. doi: 10.1038/s41420-023-01705-6 (PMC10646043; doi:10.1038/s41420-023-01705-6)
Supplement: Supplementary file 1 — Supplementary Figures and Tables [file 41420_2023_1705_MOESM1_ESM.docx]

**Supplementary Figures and Tables**

**Supplementary Figures**

**
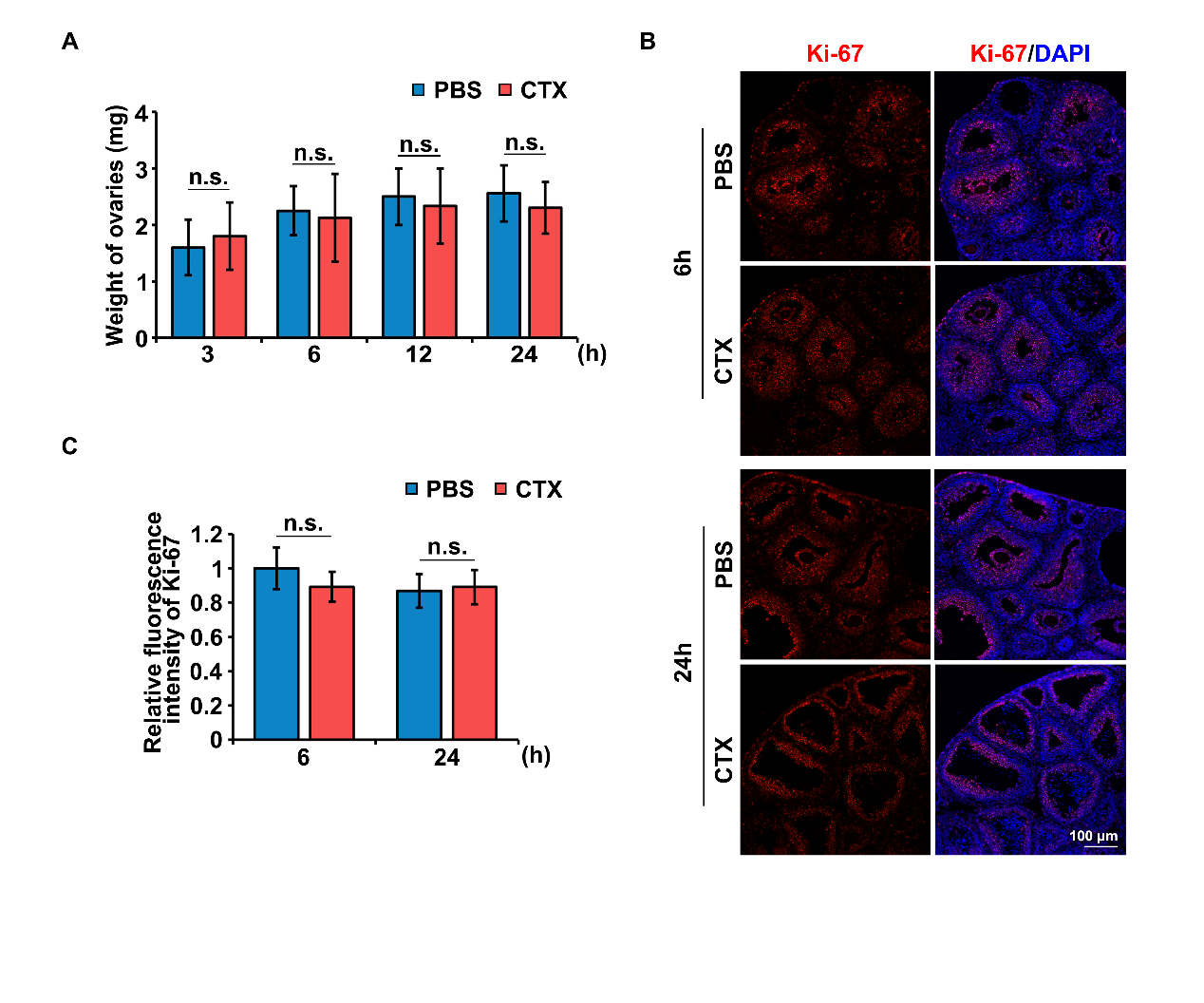
**

**Fig. S1 The effect of CTX on weight of ovary and Ki-67.** **A** Weight of the ovaries isolated from mice treated with or without CTX 24 h prior. Ovaries were collected at different time points after i.p. injection of PBS or CTX. *N* = 8 mice per time point for each group. **B** Immunofluorescence staining of ovarian sections 6 and 24 h after i.p. injection of PBS or CTX. Rabbit monoclonal antibody Ki-67 was detected using anti-rabbit IgG (red). Cell nuclei were labeled with DAPI (blue); scale bar, 100 μm; *N* = 6 ovaries from different mice per time point for each group. **C** Quantitative plots for fluorescence intensity of Ki-67. Statistical analyses were carried out using a two-tailed Student’s t-test; n.s.: non-significant; * *P* < 0.05; ** *P* < 0.01; and *** *P* < 0.001.

**Supplementary Tables**

**Supplementary Table 1: Antibody information.**

| **Protein name** | **Manufacture (catalogue number)** | **Applications (working dilution)** |
| --- | --- | --- |
| H3K27me3 | Cell Signaling (9733) | IF (1:200), WB (1:1000) |
| H2AK119ub1 | Cell Signaling (8240) | WB (1:1000) |
| H3K9me3 | Cell Signaling (5237) | IF (1:200), WB (1:1000) |
| H3K4me3 | Abcam (ab213224) | WB (1:1000) |
| Phospho-Histone H2A.X (Ser139) | Cell Signaling (9718) | WB (1:1000) |
| PARP | Cell Signaling (9532) | WB (1:1000) |
| Cleaved PARP | Cell Signaling (5625) | WB (1:1000) |
| BCL-2 | Cell Signaling (15071) | WB (1:1000) |
| Cleaved Caspase-3 (Asp175) | Cell Signaling (9661) | IF (1:200) |
| Ki-67 | Abcam (ab15580) | IF (1:200) |
| EZH2 | Cell Signaling (5246) | WB (1:1000) |
| SUZ12 | Cell Signaling (3737) | WB (1:1000) |
| H3 | Cell Signaling (9715) | WB (1:2000) |
| β-Actin | Sigma (F3165) | WB (1:5000); |
| α-Tubulin | Abcam (ab7291) | IF (1:1000) |
| β-tubulin | Cell Signaling (2125S) | WB (1:5000) |
| GAPDH | Proteintech (60004-1-Ig) | WB (1:5000) |

**Supplementary Table 2:** **Primer sequences for RT-qPCR.**

| **Primer name** | **Target Gene** | **Sequences (5′-3′)** |
| --- | --- | --- |
| *Ezh2*-F | *Ezh2* | 5’- CGAATAACAGTAGCAGACCCAG -3’ |
| *Ezh2*-R |  | 5’- TGTTTGACACCGAGAATTTGCTT -3’ |
| *Cdkn1a*-F | *Cdkn1a* | 5’- CCTGGTGATGTCCGACCTG -3’ |
| *Cdkn1a*-R |  | 5’- CCATGAGCGCATCGCAATC -3’ |
| *Eda2r*-F | *Eda2r* | 5’- ATGAGGCTGAAAAGACAGTGG -3’ |
| *Eda2r*-R |  | 5’- TCCAGGATGGAGTTAAGTGGTT -3’ |
| *Fas*-F | *Fas* | 5’- TATCAAGGAGGCCCATTTTGC -3’ |
| *Fas*-R |  | 5’- TGTTTCCACTTCTAAACCATGCT -3’ |
| *Ccng1*-F | *Ccng1* | 5’- ACAACTGACTCTCAGAAACTGC -3’ |
| *Ccng1*-R |  | 5’- CATTATCATGGGCCGACTCAAT -3’ |
| *Gadd45g*-F | *Gadd45g* | 5’- GGGAAAGCACTGCACGAACT -3’ |
| *Gadd45g*-R |  | 5’- AGCACGCAAAAGGTCACATTG -3’ |
| *Alox5-*F | *Alox5* | 5’- ACTACATCTACCTCAGCCTCATT -3’ |
| *Alox5*-R |  | 5’- GGTGACATCGTAGGAGTCCAC -3’ |
| *Egr4*-F | *Egr4* | 5’- TATCCTGGAGGCGACTTCTTG -3’ |
| *Egr4*-R |  | 5’- AGATGCCAGACATGAGGTTGA -3’ |
| *Klf4*-F | *Klf4* | 5’- GTGCCCCGACTAACCGTTG -3’ |
| *Klf4*-R |  | 5’- GTCGTTGAACTCCTCGGTCT -3’ |
| *Btg2*-F | *Btg2* | 5’- ATGAGCCACGGGAAGAGAAC -3’ |
| *Btg2*-R |  | 5’- GCCCTACTGAAAACCTTGAGTC -3’ |
| *Actb*-F | *Actb* | 5′- GCTCTTTTCCAGCCTTCCTT -3′ |
| *Actb*-R |  | 5′- GTACTTGCGCTCAGGAGGAG -3′ |
