# Supplementary material for: The EZH2-H3K27me3 axis modulates aberrant transcription and apoptosis in cyclophosphamide-induced ovarian granulosa cell injury
Source: Cell Death Discov. 2023 Nov 14;9:413. doi: 10.1038/s41420-023-01705-6 (PMC10646043; doi:10.1038/s41420-023-01705-6)

Uncropped Western Blot

Figure in paper	Target / Protein	Blot name	Band #
Figure 1a	γH2AX	Blot1_Fig1a_γH2AX	all
Figure 1a	cPARP	Blot2_Fig1a_ cPARP	all (the upper lane)
Figure 1a	PARP	Blot3_Fig1a_ PARP	all
Figure 1a	BCL2	Blot4_Fig1a_BCL2	all
Figure 1a	GAPDH	Blot5_Fig1a_GAPDH	all
Figure 1a	β-tubulin	Blot6_Fig1a_Tubulin	all
Figure 2a	H3K27me3	Blot7_Fig2a_ H3K27me3	all
Figure 2a	H2AK119ub1	Blot8_Fig2a_ H2AK119ub1	all
Figure 2a	H3K9me3	Blot9_Fig2a_ H3K9me3	all
Figure 2a	H3K4me3	Blot10_Fig2a_ H3K4me3	all
Figure 2a	H3	Blot11_Fig2a_ H3	all
Figure 2a	EZH2	Blot12_Fig2a_ EZH2	all
Figure 2a	SUZ12	Blot13_Fig2a_SUZ12	all
Figure 2a	GAPDH	Blot14_Fig2a_ GAPDH	all
Figure 2a	β-tubulin	Blot15_Fig2a_ Tubulin	all
Figure 2e	EZH2	Blot16_Fig2e_ EZH2	6-11
Figure 2e	H3K27me3	Blot17_Fig2e_ H3K27me3	6-11
Figure 2e	γH2AX	Blot18_Fig2e_γH2AX	3-8
Figure 2e	β-actin	Blot19_Fig2e_Actin	6-11

Figure 2e	H3	Blot20_Fig2e_H3	all
Figure 2g	EZH2	Blot21_Fig2g_ EZH2	1-2
Figure 2g	H3K27me3	Blot22_Fig2g_ H3K27me3	1-2
Figure 2g	γH2AX	Blot23_Fig2g_γH2AX	1-2
Figure 2g	β-actin	Blot24_Fig2g_Actin	1-2
Figure 2g	H3	Blot25_Fig2g_H3	1-2
Figure 5b	cPARP	Blot26_Fig5b_ cPARP	all
Figure 5b	PARP	Blot27_Fig5b_ PARP	all
Figure 5b	γH2AX	Blot28_Fig5b_γH2AX	all
Figure 5b	H3K27me3	Blot29_Fig5b_ H3K27me3	all
Figure 5b	β-tubulin	Blot30_Fig5b_Tubulin	all
Figure 5b	β-actin	Blot31_Fig5b_Actin	4-6
Figure 5b	H3	Blot32_Fig5b_ H3	all

Blot1_Fig1a_γH2AX

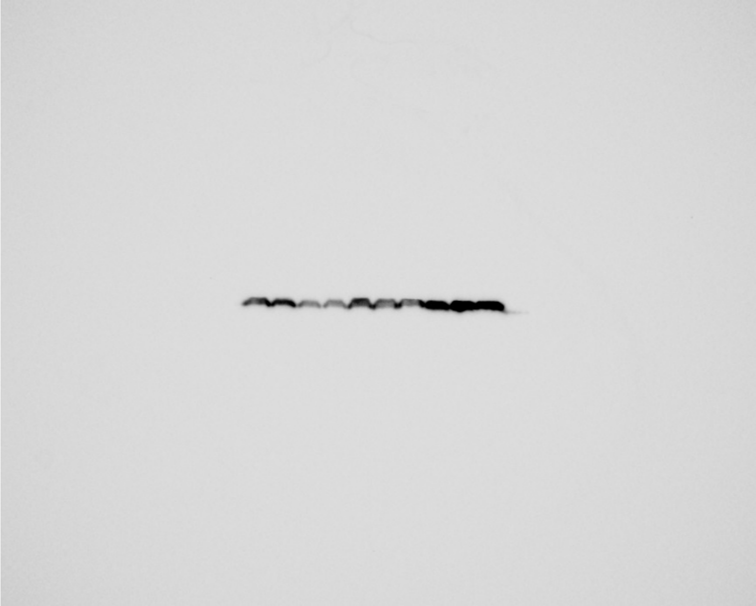

Blot2_Fig1a_ cPARP

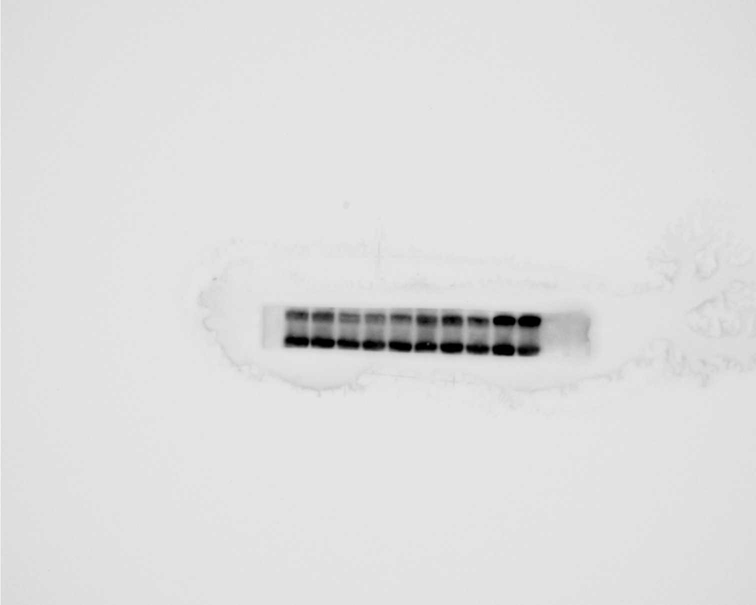

Blot3_Fig1a_ PARP

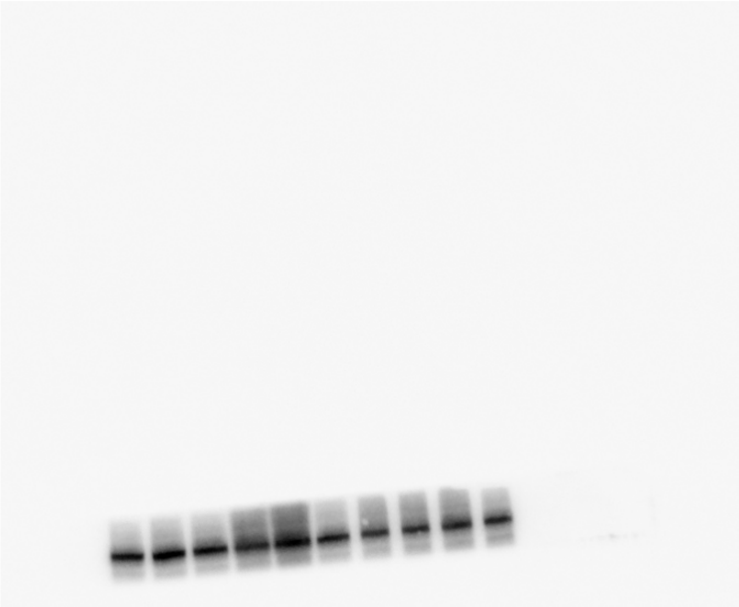

Blot4_Fig1a_BCL2

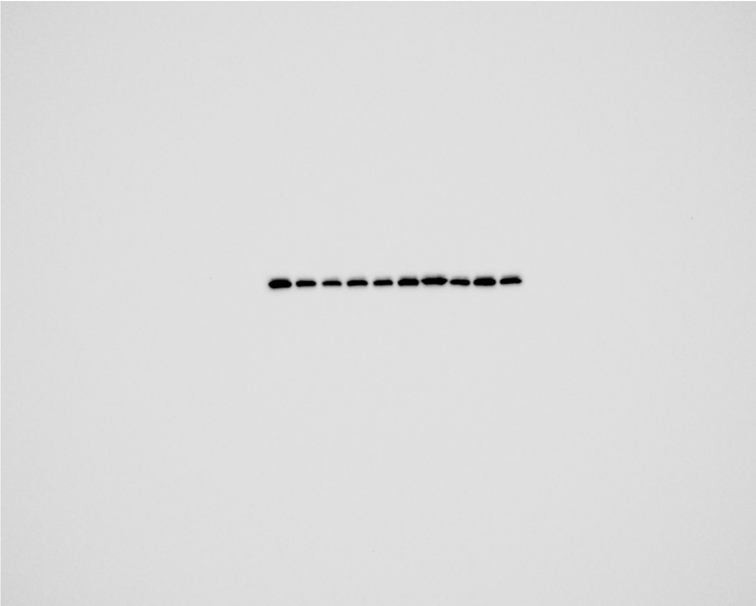

Blot5_Fig1a_GAPDH

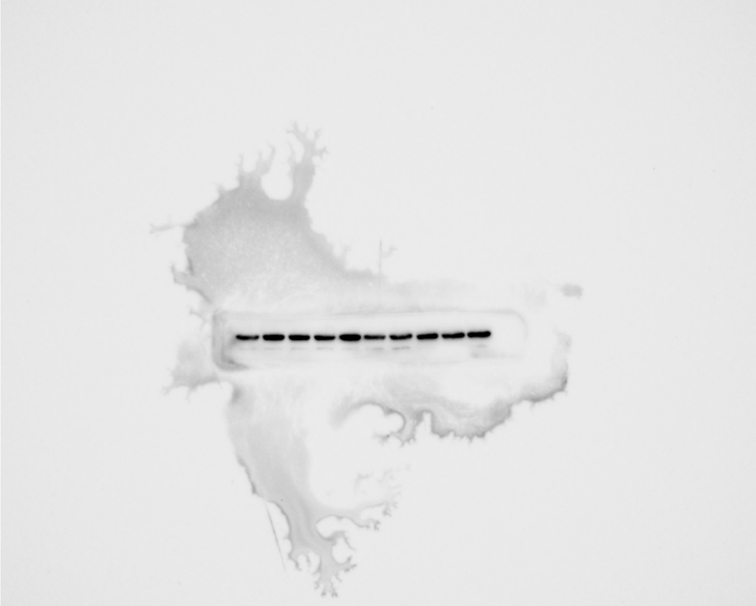

Blot6_Fig1a_Tubulin

Blot7_Fig2a_ H3K27me3

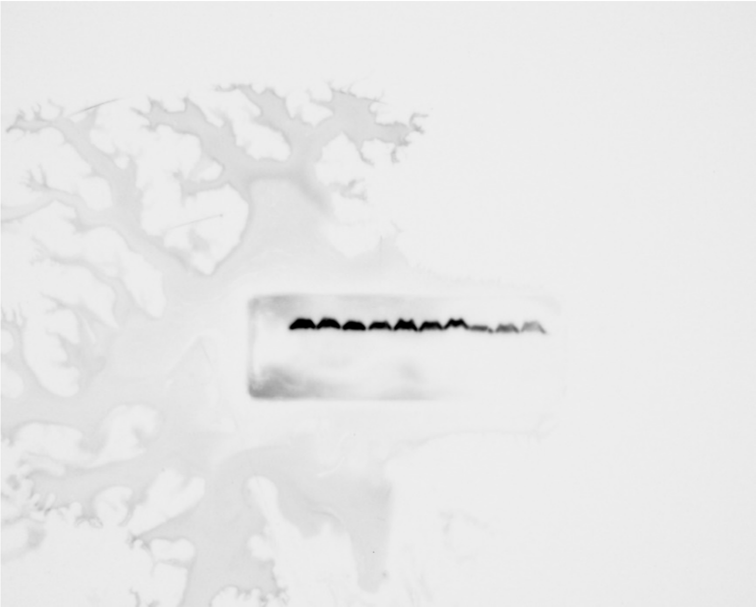

Blot8_Fig2a_ H2AK119ub1

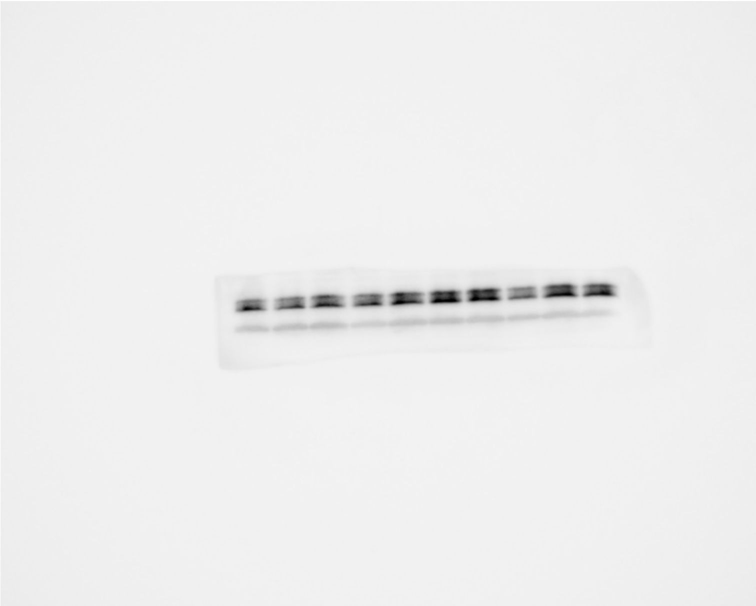

Blot9_Fig2a_ H3K9me3

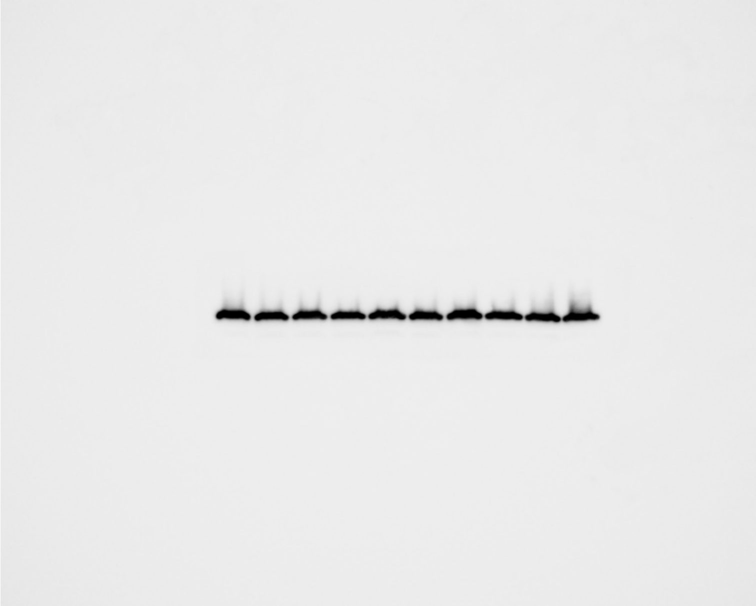

Blot10_Fig2a_ H3K4me3

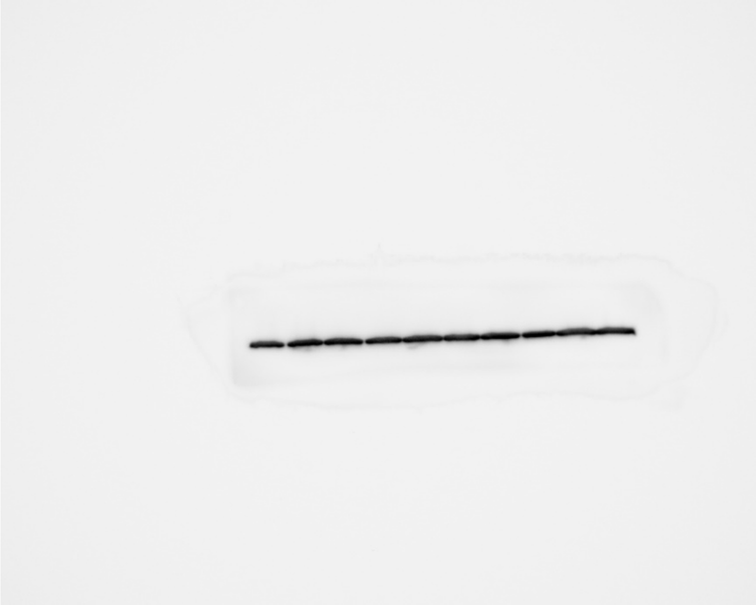

Blot11_Fig2a_ H3

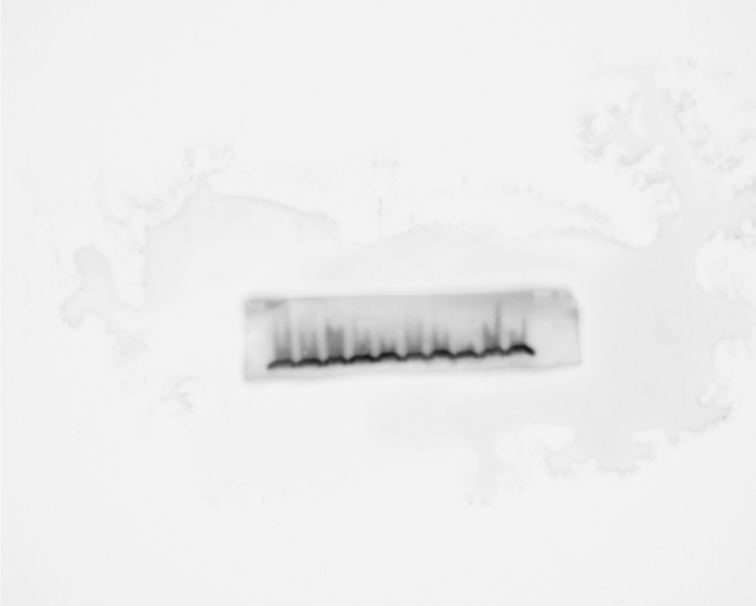

Blot12_Fig2a_ EZH2

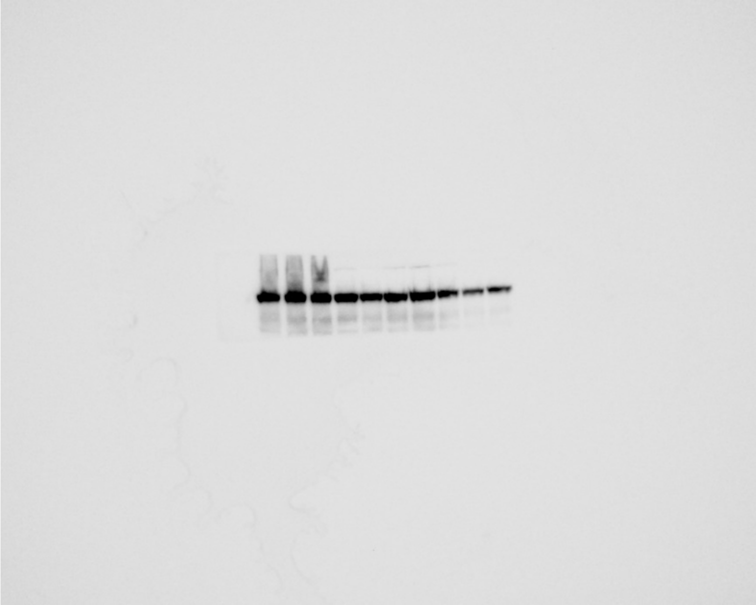

Blot13_Fig2a_SUZ12

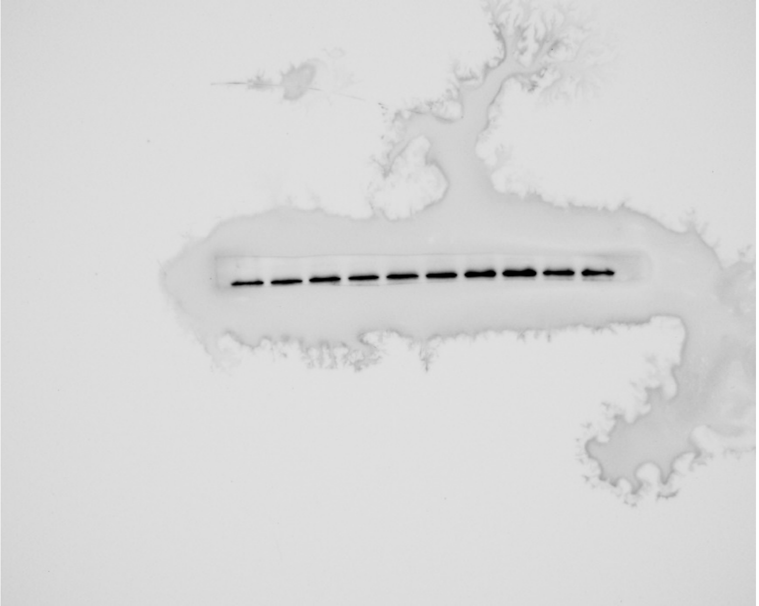

Blot14_Fig2a_ GAPDH

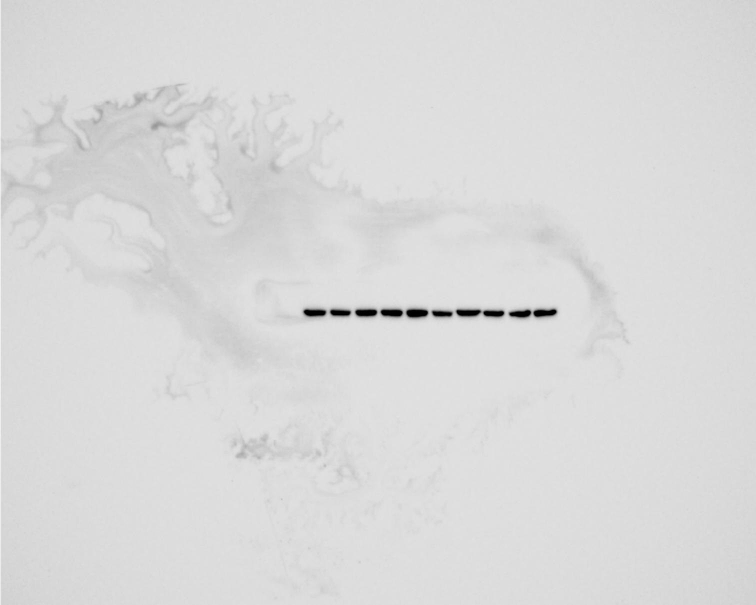

Blot15_Fig2a_ Tubulin

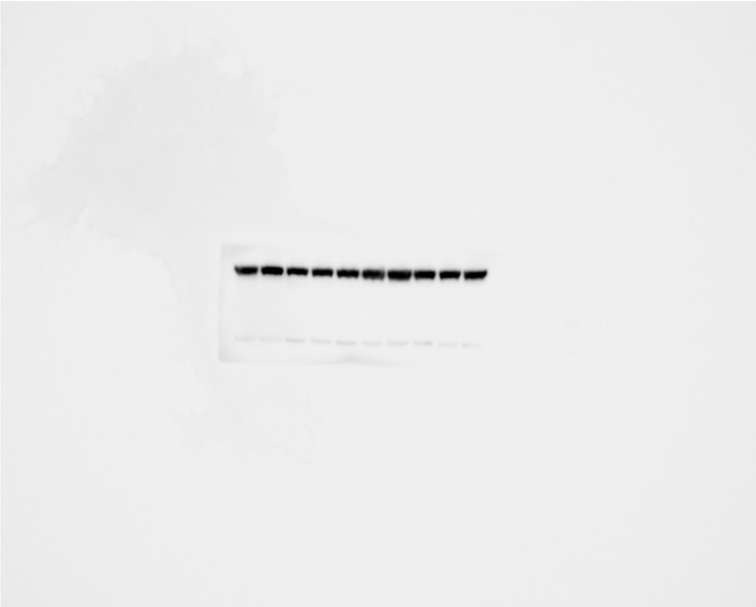

Blot16_Fig2e_ EZH2

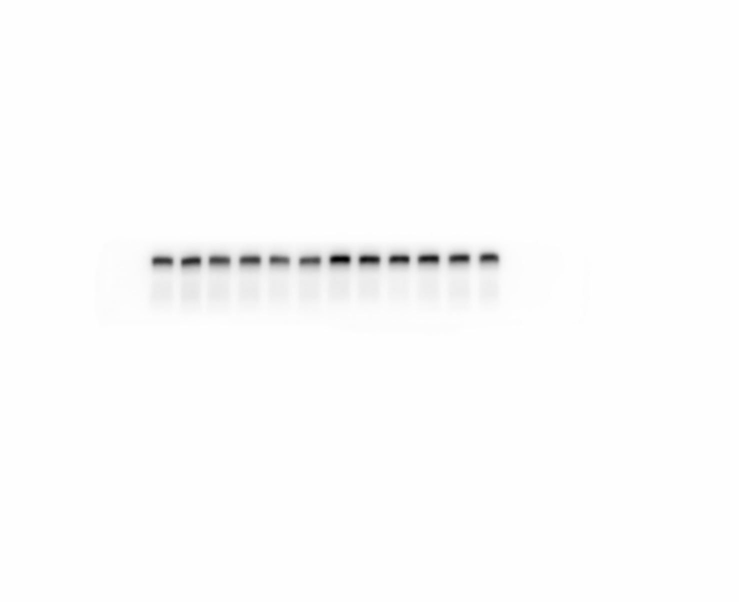

Blot17_Fig2e_ H3K27me3

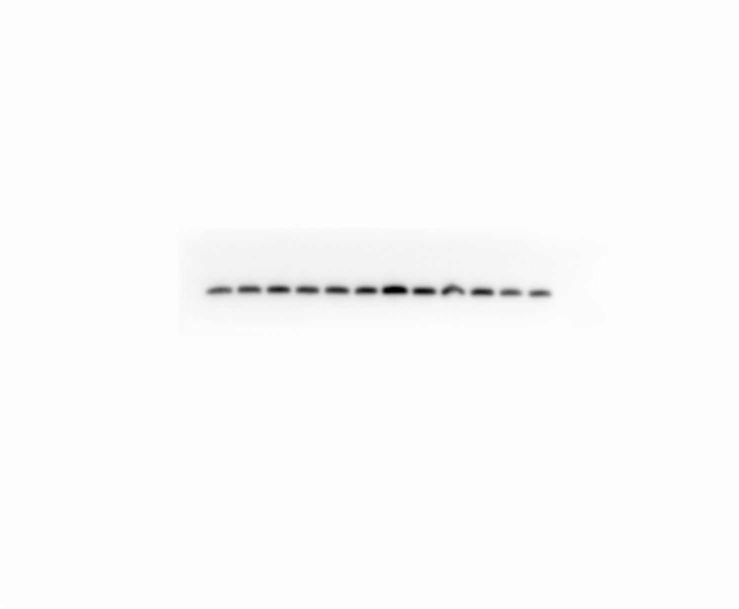

Blot18_Fig2e_γH2AX

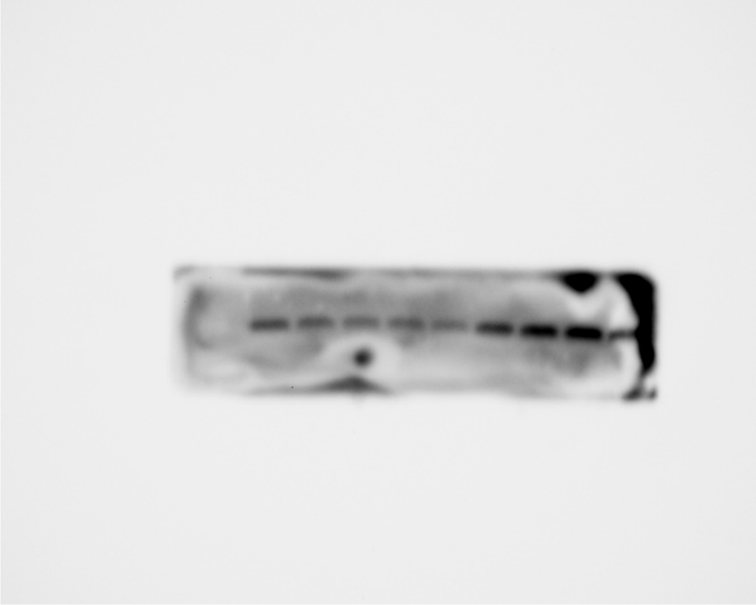

Blot19_Fig2e_Actin

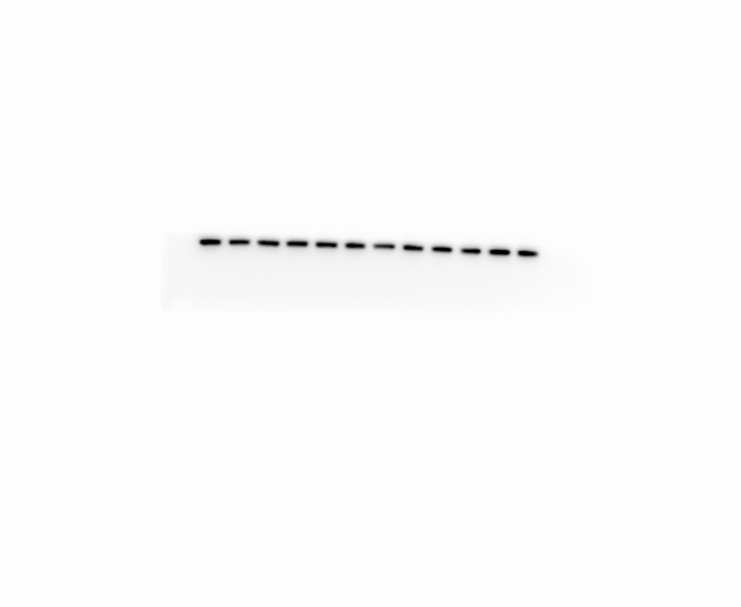

Blot20_Fig2e_H3

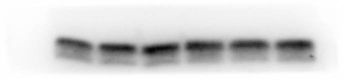

Blot21_Fig2g_ EZH2

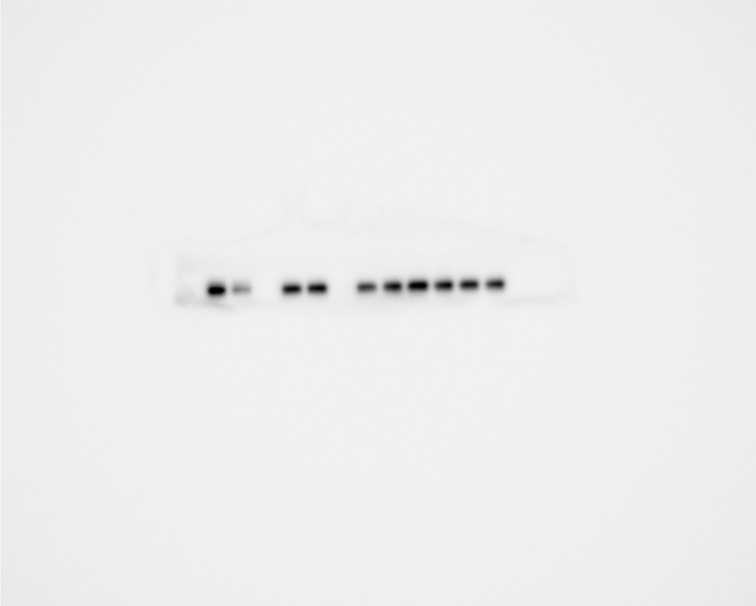

Blot22_Fig2g_ H3K27me3

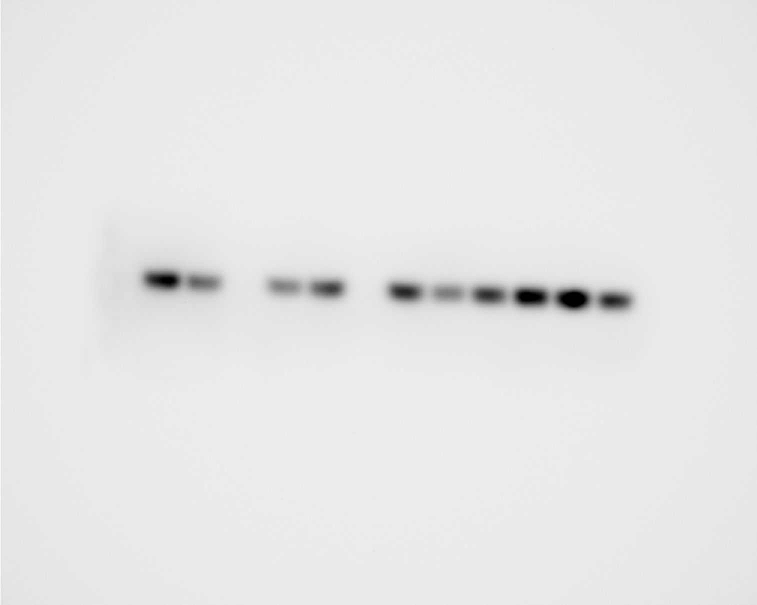

Blot23_Fig2g_γH2AX

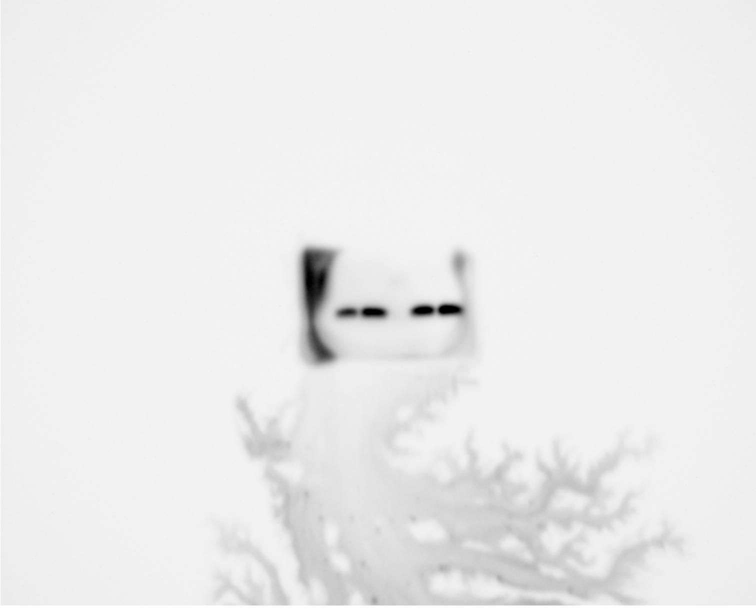

Blot24_Fig2g_Actin

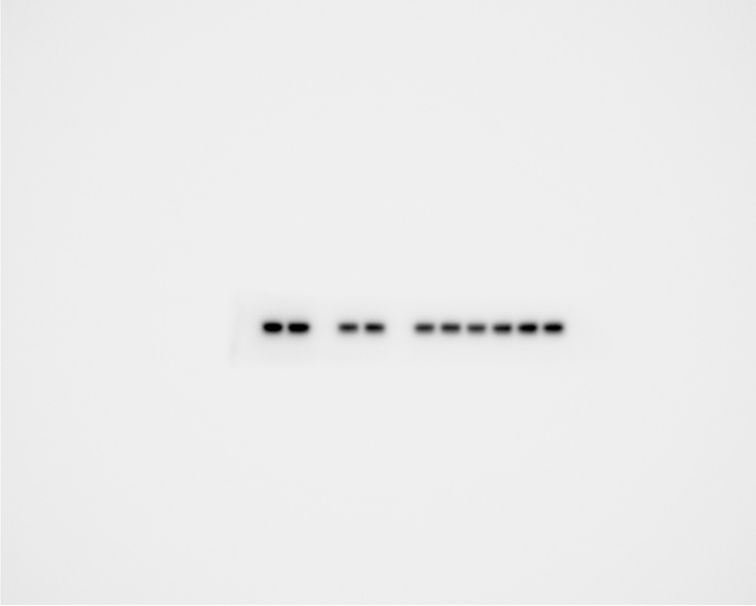

Blot25_Fig2g_H3

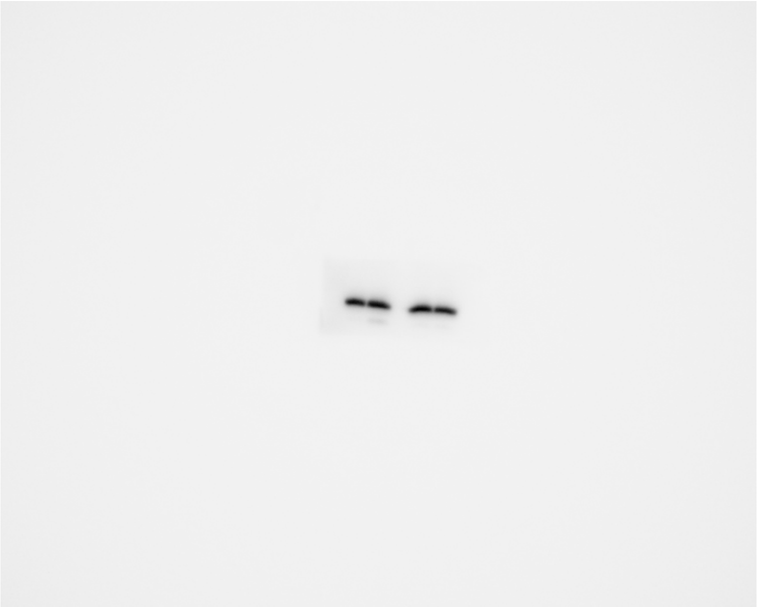

Blot26_Fig5b_ cPARP

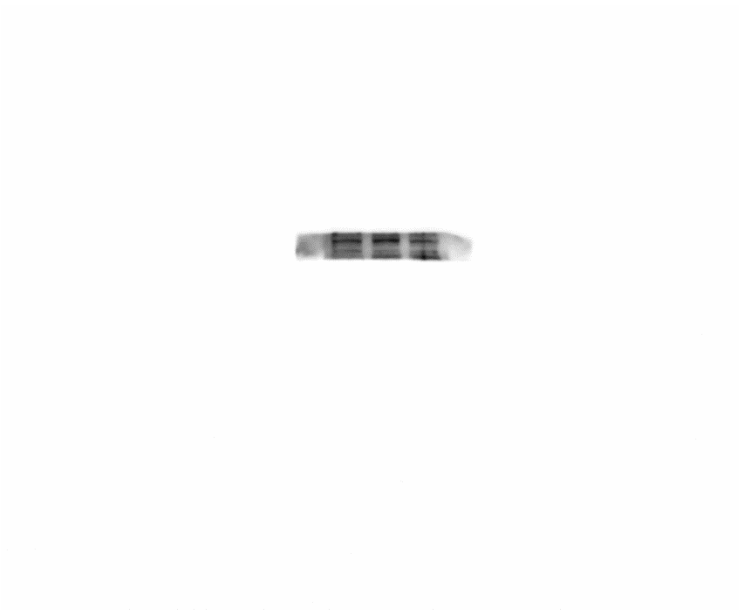

Blot27_Fig5b_ PARP

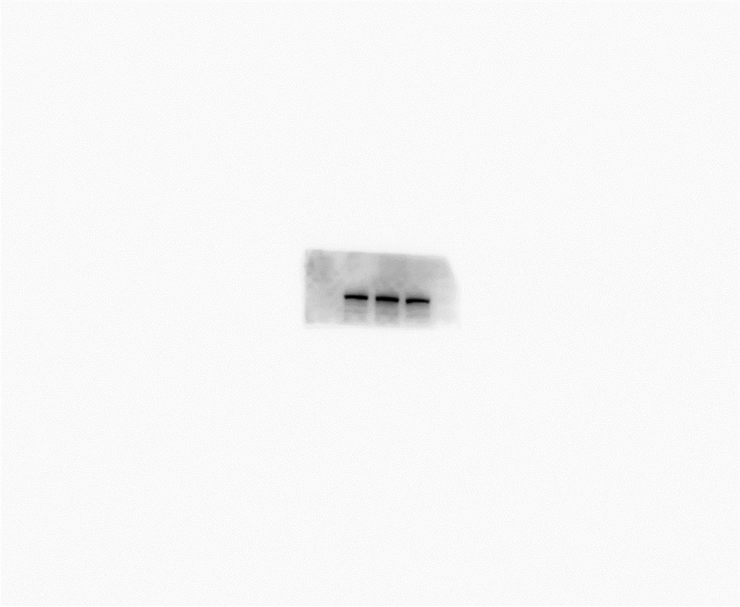

Blot28_Fig5b_γH2AX

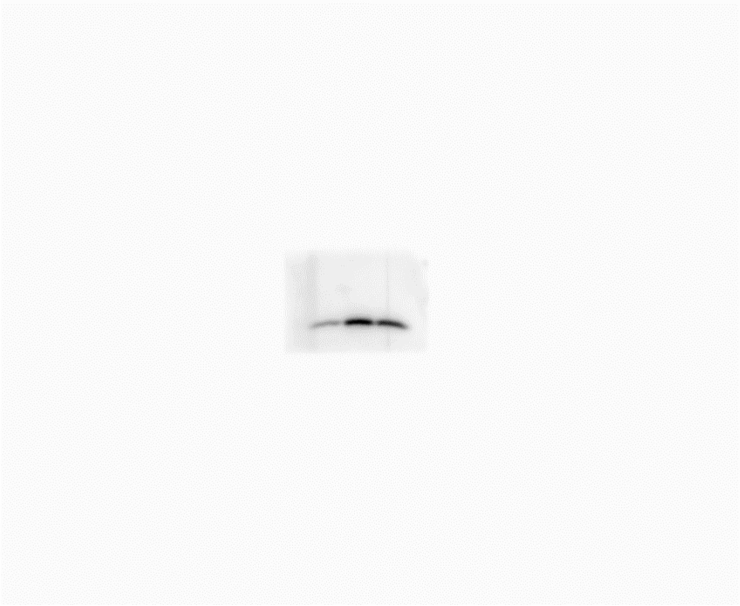

Blot29_Fig5b_ H3K27me3

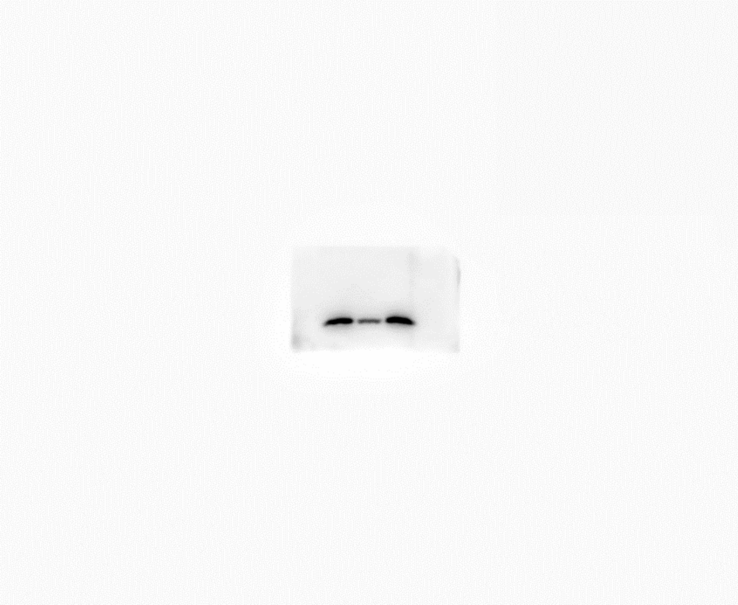

Blot30_Fig5b_Tubulin

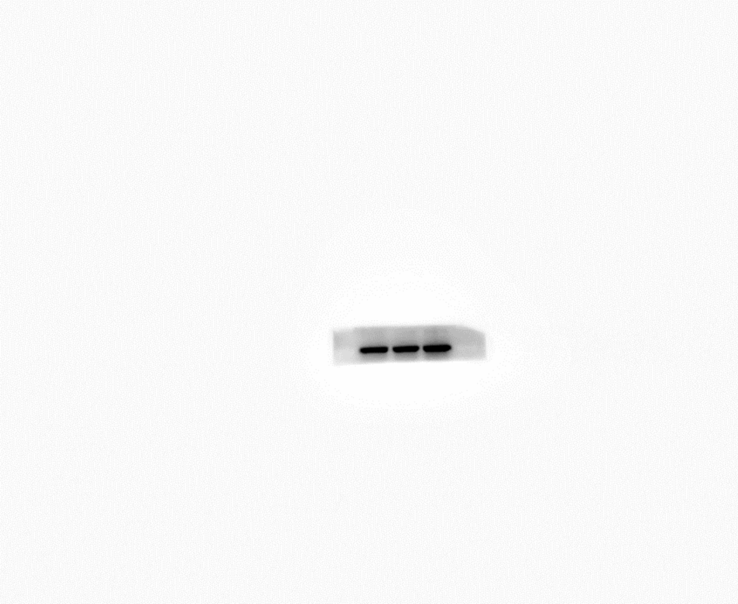

Blot31_Fig5b_Actin

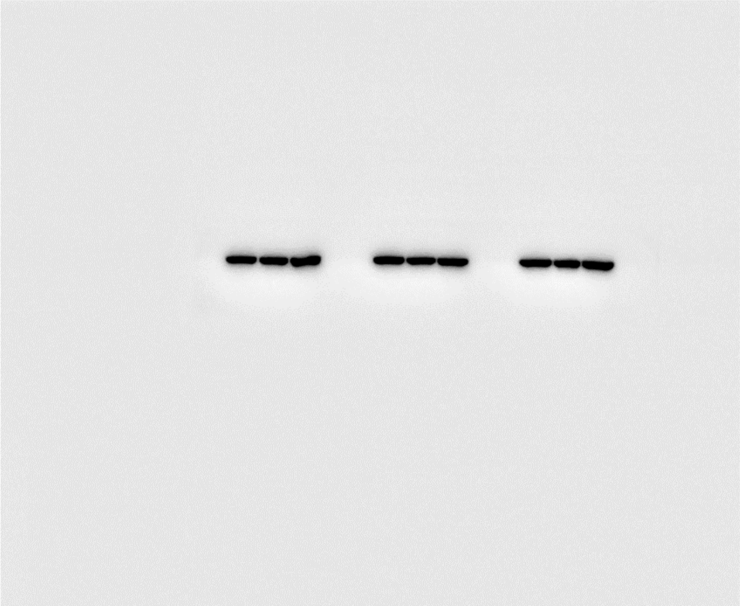

Blot32_Fig5b_ H3

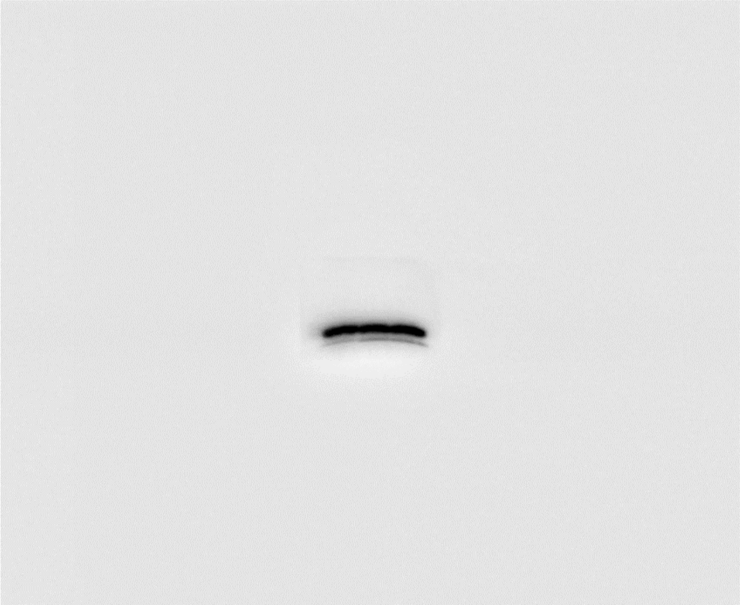

Supplement: Supplementary file 2 — Uncropped western blot [file 41420_2023_1705_MOESM2_ESM.pdf]
